# Therapeutic targets for HIV-1 infection in the host proteome

**DOI:** 10.1186/1742-4690-2-20

**Published:** 2005-03-21

**Authors:** Winnie S Liang, Anil Maddukuri, Tanya M Teslovich, Cynthia de la Fuente, Emmanuel Agbottah, Shabnam Dadgar, Kylene Kehn, Sampsa Hautaniemi, Anne Pumfery, Dietrich A Stephan, Fatah Kashanchi

**Affiliations:** 1 Department of Biochemistry and Molecular Biology, George Washington University School of Medicine, Washington, DC 20037, USA; 2 Neurogenomics Division, Translational Genomics Research Institute, Phoenix, AZ 85004, USA; 3 Institute for Genetic Medicine, Johns Hopkins Medical School, Baltimore, MD 21205, USA; 4 Institute of Signal Processing, Tampere University of Technology, PO Box 553, 33101, Tampere, Finland; 5 The Institute for Genomic Research, TIGR, Rockville, MD 20850, USA

## Abstract

**Background:**

Despite the success of HAART, patients often stop treatment due to the inception of side effects. Furthermore, viral resistance often develops, making one or more of the drugs ineffective. Identification of novel targets for therapy that may not develop resistance is sorely needed. Therefore, to identify cellular proteins that may be up-regulated in HIV infection and play a role in infection, we analyzed the effects of Tat on cellular gene expression during various phases of the cell cycle.

**Results:**

SOM and k-means clustering analyses revealed a dramatic alteration in transcriptional activity at the G1/S checkpoint. Tat regulates the expression of a variety of gene ontologies, including DNA-binding proteins, receptors, and membrane proteins. Using siRNA to knock down expression of several gene targets, we show that an Oct1/2 binding protein, an HIV Rev binding protein, cyclin A, and PPGB, a cathepsin that binds NA, are important for viral replication following induction from latency and *de novo *infection of PBMCs.

**Conclusion:**

Based on exhaustive and stringent data analysis, we have compiled a list of gene products that may serve as potential therapeutic targets for the inhibition of HIV-1 replication. Several genes have been established as important for HIV-1 infection and replication, including Pou2AF1 (OBF-1), complement factor H related 3, CD4 receptor, ICAM-1, NA, and cyclin A1. There were also several genes whose role in relation to HIV-1 infection have not been established and may also be novel and efficacious therapeutic targets and thus necessitate further study. Importantly, targeting certain cellular protein kinases, receptors, membrane proteins, and/or cytokines/chemokines may result in adverse effects. If there is the presence of two or more proteins with similar functions, where only one protein is critical for HIV-1 transcription, and thus, targeted, we may decrease the chance of developing treatments with negative side effects.

## Background

With the rapid emergence of the HIV-1 and AIDS pandemic, tremendous effort has been directed towards development of effective treatments and vaccines. Currently, HAART is the only therapeutic option available for seropositive and symptomatic individuals, and is comprised of targeted inhibitors of HIV-1 reverse transcriptase (NNRTIs and NRTIs) and/or protease (PI) and the newly FDA approved gp41-inhibitor Fuzeon/T20 [1]. Though HAART is effective in prolonging life, its use, coupled with other factors, engenders rapid development of multiple drug-resistant strains. Therefore, the comprehensive elucidation of HIV-1-mediated effects on host cellular networks is urgently needed for rational therapeutic targets. HIV-1 infection, pathogenesis, and AIDS development are largely due to the various retroviral structural, regulatory, and accessory proteins, but more importantly due to efficient 'hijacking' of cell regulatory machineries, including the differential expression of receptors, transcription, mRNA processing, and translation factors. While there has been much research on the effects of viral proteins on host cellular pathways, HIV-1 Tat appears to be the most critical for viral transcription and replication.

HIV-1 Tat is absolutely required for productive, high titer viral replication. Though its sequence and a number of its functions have been uncovered, there is still much to learn about its replication-driven and pathogenic mechanisms, including the identification and characterization of Tat-regulated *cellular *genes. With the advent of microarray technologies, it is now possible to assay the entire human genome for the effects of a single gene product, viral infection, or drug treatment. Many laboratories have previously demonstrated the effects of Tat on cell cycle-regulated transcription [2-4]. The finding that Tat activates gene expression at both the G_1 _(TAR-dependent) and G_2 _(TAR-independent) phases of the cell cycle demonstrates a concerted effort by Tat to take full advantage of cell cycle regulatory checkpoints. These findings prompted us to explore the effects of constitutive Tat expression on the expression profile of 1,200 host cellular genes in HIV-1 infected unsynchronized cells [5]. We observed that while the majority of cellular genes were down-regulated, especially those with intrinsic receptor tyrosine kinase activity, numerous S phase and translation-associated genes were up-regulated. These findings and the fact that inducing a G_1_/S block on infected cells dramatically reduces viral transcription and progeny formation [6-8], prompted us to follow and elucidate the effects of Tat on the host transcriptional profile throughout the entire cell cycle.

Here, we report the HIV-1 Tat-mediated effects on the host expression profile relative to the cell cycle. We first performed microarray experiments in unsynchronized Tat-expressing cells compared to empty vector-transfected cells. We subsequently performed similar experiments in synchronized cells at the G_1_/S and G_2_/M phase boundaries. Cells were then collected at 0 h, 3 h, 6 h, and 9 h post-release per treatment corresponding to a specific cell cycle stage, and cytoplasmic RNA was isolated for microarray analysis. After microarray analysis using the Affymetrix U95Av2 gene chip, we found a wide variety of gene ontologies that were affected by Tat through cell cycle progression. We confirmed that Tat differentially regulates the expression of a variety of genes at different phases of the cell cycle, with an overall inhibition of the cellular transcription profile. Using siRNA technology to 'knock-down' protein expression, we screened several of these genes as possible therapeutic targets for inhibition of HIV-1 replication. We generated a comprehensive list of Tat-induced genes at each cell cycle phase, particularly the G_1_/S phase transition, and expanded the list of Tat-regulated cellular proteins and potential therapeutic targets.

## Results and Discussion

### Microarray design and analysis

**Figure 1:**
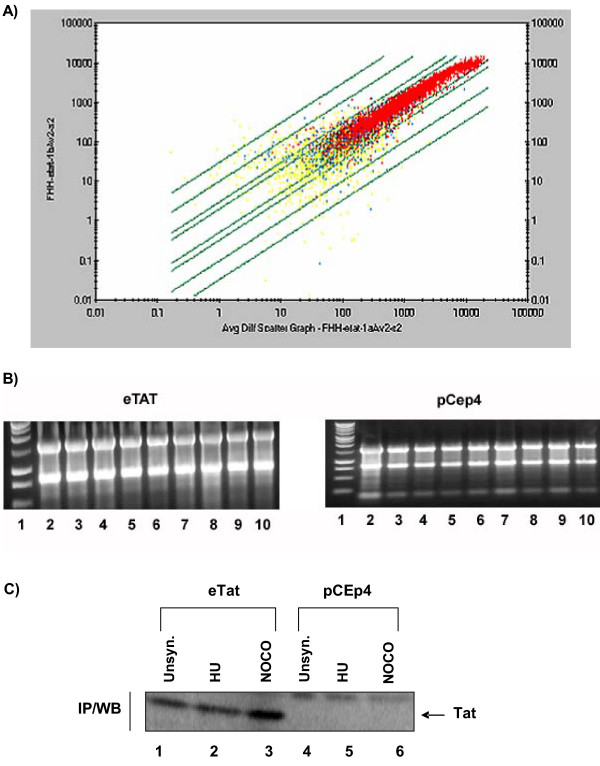
**Cross-validity of Tat samples and RNA isolation. **(A) Cross-validity of the duplicate Tat samples analyzed. With a total of 32 gene chips, we analyzed the reliability of the gene chip samples relative to their respective replicate. The scatter graph logarithmically plots the signal intensity values of probe sets for one sample against those for a sample replicate. Each graph point indicates a common probe set between the two data sets and the value is determined by the intersection of the x and y values for that probe set. 2-fold, 3-fold, and 10-fold change lines are defined by the following equations: y = 2x and y = 1/2x, y = 3x and y = 1/3x, y = 10x and y = 1/10x, y = 30x and y = 1/30x. Yellow spots represent probes with absent-absent, absent-marginal, marginal-absent, and marginal-marginal detection calls on sample replicates. Blue spots represent those with absent-present, present-absent, marginal-present, and present-marginal calls, while red spots represent probe sets with present-present detection calls. (B) Cytoplasmic RNA was isolated from all experimental and corresponding control samples, and quantitated by UV spectrophotometric analysis; 3 μg was run on a 1% agarose gel for visual inspection. (C) IP/Westerns for Tat protein. Lanes 1–3 are from eTat extracts and Lanes 4–6 are from control pCep4 cells; unsynchronized cells are shown in Lanes 1 and 4.

To understand which cellular genes were affected by Tat, we analyzed the transcription profile of ~12,000 gene transcripts using the Affymetrix U95Av2 gene chip. Cells were either transfected with the eTat plasmid or a pCep4 control vector. We chose to perform experimental and control conditions in duplicate to account for inter-chip variability. Figure 1A illustrates the cross-validity of the duplicate synchronized cell cycle experiments run for the eTat samples. The scatter plot graph logarithmically plots the probe set signal intensity values from the first experiment against those from the second experiment (average R^2 ^value = 0.912). Yellow spots represent gene probes with absent or marginal calls and the blue spots correspond to probes with present and marginal calls. Blue spots show less correlation and the yellow spots indicate the lowest level of correlation. Red spots represent those probes that displayed present calls in both experiments and thus demonstrate the highest level of correlation. The fold change lines indicate two-fold, three-fold, and ten-fold changes. Figure 1A shows the correlation of signal and detection values between the two experiments for each probe set, as well as the reliability of one dataset compared to its replicate. Similar results were observed for this analysis between the duplicate control pCep4 samples (data not shown). Though previous microarray experiments performed by us and others have used total nuclear and cytoplasmic RNA, we chose to isolate only cytoplasmic RNA because nuclear RNA would include RNAs that have been improperly spliced, or uncapped, and may have contain inappropriate poly-A tails, while cytoplasmic RNAs would yield almost a complete RNA population that has been properly processed prior to nuclear export and translation. As seen in Figure 1B, the RNA samples for both experiments show good RNA integrity with defined 18S and 28S bands.

We first studied the effects of constitutive Tat expression on the host cell transcription profile in unsynchronized cells and then relative to the cell cycle phases. Initially, a heterogenous cell population of Tat-expressing cells was compared to one expressing the pCep4 vector to create a global Tat-induced transcription profile. In the latter experiment, samples were treated with either hydroxyurea (Hu) or nocodazole (Noco) for 18 h to obtain either a G_1_/S or G_2_/M block, respectively. Cells blocked with Hu were 60% at G_1_, 35% at S, and 5% at the G_2_/M phase, while cells blocked with Noco were 6% at G_1_, 24% at S, and 70% at the G_2_/M phase (data not shown). Following cell cycle arrest, cells were washed and released in complete media. The 0 h time point following Hu treatment is representative of the G_1_/S phase of the cell cycle, while the 3 h, 6 h, and 9 h time points correspond to the early S, late S, and G_2 _phases, respectively. Noco, a G_2_/M phase blocker, was added to the cell populations and the cells were likewise released. Samples were taken at the 0 h, 3 h, 6 h, and 9 h time points to obtain cells in the M and early, middle, and late G_1 _phases, respectively. Immunoprecipitation and western blot analysis of tat protein were also carried out to verify the presence of tat in the unsynchronized and synchronized Tat-expressing cells and those expressing the pCep4 vector (Figure 1C). Thus, we obtained and analyzed the HIV-1 Tat-induced transcription profile at every cell cycle stage. All cell cycle phase populations were confirmed using FACS analysis as previously shown [2].

### Gene expression analysis in unsynchronized Tat-expressing cells

**Figure 2:**
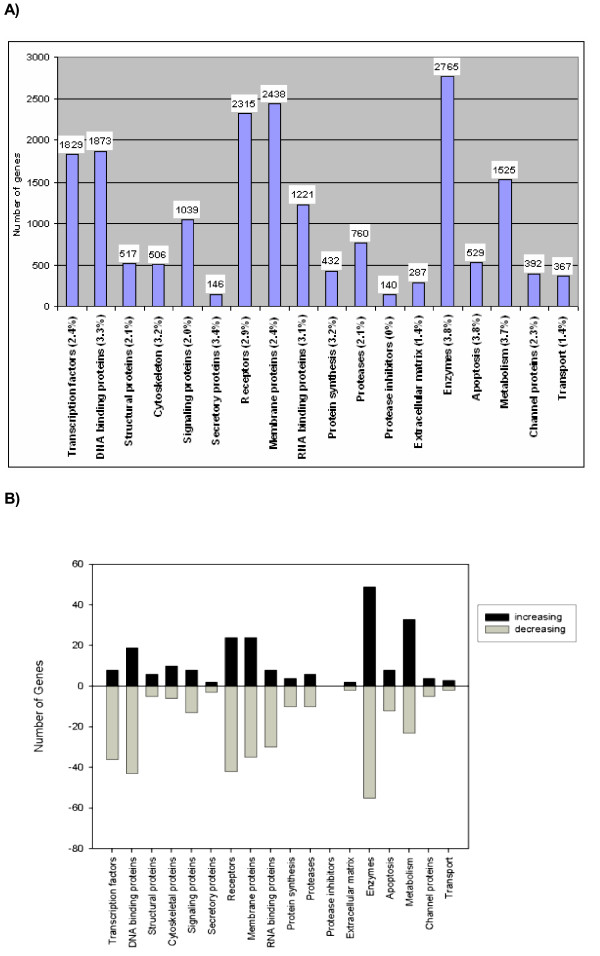
**Gene ontologies present on the human U95Av2 chip and those specifically induced by Tat. **(A) The U95Av2 gene chip was surveyed to determine the ontology of genes represented on the chip, as well as the corresponding number of genes belonging to each category. The percentages next to each classification correspond to the percentage of genes affected by Tat. (B) HIV-1 Tat-induced/repressed genes in an unsynchronized HeLa-eTat cell population. The number of genes induced/repressed by Tat, as well as the various classifications, is shown.

We analyzed the differential gene expression of a Tat-expressing cell population relative to that of a control population. This microarray analysis consisted of looking at ~12,000 genes in unsynchronized cells to ascertain the global effect of HIV-1 Tat-mediated transcriptional regulation on the host cell genome. Overall, we observed Tat-induced/-repressed differential expression of 649 genes (~5% of genes screened) belonging to a wide variety of gene ontologies (Figure 2A). Figure 2B depicts gene ontologies for genes showing increased/decreased expression between the eTat and pCep4 samples. A few genes were represented as belonging to a variety of classifications and were placed into multiple categories. We observed the greatest effect (~3%) of Tat on genes encoding for cellular enzymes; secretory, metabolic, and apoptotic pathways; and RNA binding, DNA binding, cytoskeletal, protein synthesis, and receptor proteins, while the other gene ontologies were less affected by Tat expression. We also observed that ~60% of the Tat affected genes were down-regulated. These findings are consistent with the previously published results by us and other laboratories [5,9,10].

### HIV-1 Tat-induced transcription profile

**Figure 3:**
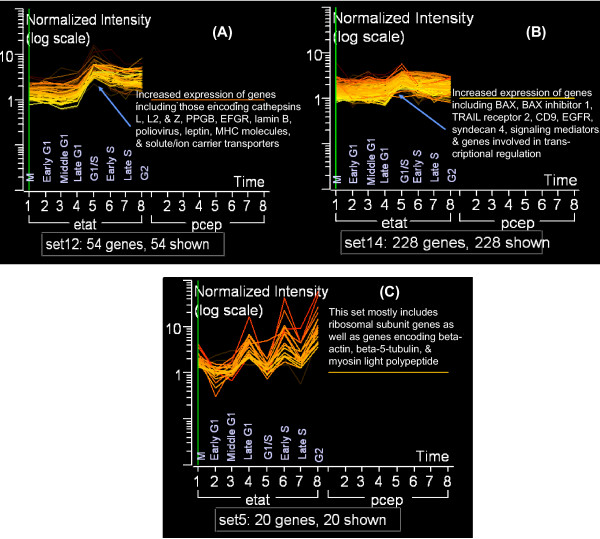
**K-Means clustering analysis of Tat-induced genes. **The temporal differential gene expression in Tat cells was compared to respective control samples and analyzed using the k-means clustering algorithm. The coordinated expression profiles are representative of the 32 chips analyzed (16 eTat and 16 pCep4). The y-axis represents the log scale of the normalized intensity of the genes shown (data was normalized against the corresponding control samples). The x-axis corresponds to the various cell cycle phases: 1) M phase, 2) early G_1_, 3) middle G_1_, 4) late G_1_, 5) G_1_/S, 6) early S, 7) late S, and 8) G_2_. Fifteen clusters were found based on the parameters used [see Additional File 7] and three are shown in 3A-C. Figure 3A shows altered genes at the G1/S for cathepsins, and various cellular receptors, while Figure 3B shows a close-up of apoptotic regulated genes, signal transduction and transcription factors. Figure 3C shows genes that dramatically oscillate at every stages of cell cycle in Tat expressing cells, including ribosome and actin/cytoskeleton genes.

**Figure 4:**
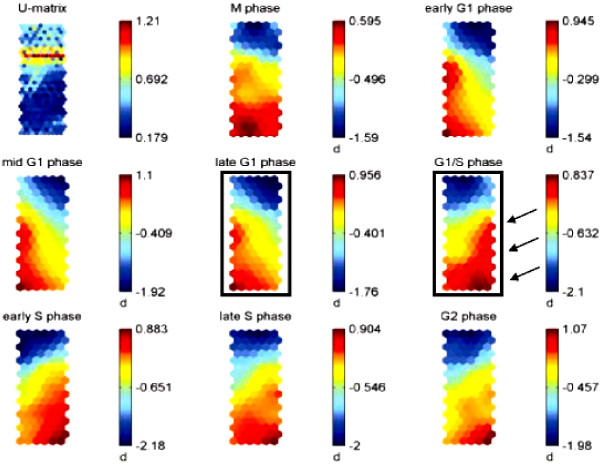
**Temporal SOM analysis of HIV-1 Tat-induced cellular genes in synchronized Tat cells. **3 separate filters were applied to remove genes that did not display at least a 1.5, 2, or 3-fold change at each time point analyzed in the 16 eTat chips (see Methods); each filter produced a discrete dataset that was applied to SOM analysis. The third and most restrictive dataset is shown here. Genes that were significantly up (red) and down-regulated (blue) are shown. The U-matrix identifies which genes are similar to each other in terms of expression profile (blue) separated by a "boundary" (red). This SOM graph contains 17 rows and 6 columns of neurons, represented as coordinates. The arrows adjacent to the G_1_/S SOM indicate those genes significantly up-regulated during this transition and S phase, and those that show decreased expression in the G_1 _phase.

Using a two-fold threshold to constrain our gene lists to those genes only significantly induced by Tat, we observed many genes that were expressed during all cell cycle phases, with fewer genes that were exclusive to only one cell cycle phase. This can be seen in both the self-organizing maps (SOMs) and k-means analysis graphs [Figures 4 and 3, respectively & Additional Files 5, 6, and 7]. In the 3 sets of SOMs generated using three separate filtering rules, we observed many genes that were relatively consistent in their expression patterns through most cell cycle phases. This was also evident in the k-means graphs that contain gene clusters whose expression was relatively linear [see Additional File 7: sets 1, 10, 11, and 14]. In the k-means analysis, the y-axis represents the normalized intensity values for the genes analyzed and the x-axis contains two sets of eight time points for each condition. K-means clustering allows for the elucidation of those genes with similar temporal expression profiles. As shown in [Additional File 7], the various graphs correspond to separate clusters of genes whose expression is similar in Tat-expressing cells relative to cell cycle progression.

Based on the k-means clustering methods, we observed a coordinated up-regulation of 228 genes during the G_1_/S phase transition in set 14 (Figure 3B) and 54 genes in set 12 (Figure 3A). On the other hand, set 5 (Figure 3C) displays genes whose expression peaks at different time points in the cell cycle, but are specifically down-regulated at the G_1_/S boundary. Set 12 (Figure 3A) was very similar to the results seen with the G_1_/S SOM (Figure 4), in which genes were up-regulated at the G_1_/S phase and continued to be highly expressed until the G_2 _phase. Set 12 illustrates the increased expression of various cathepsins (L, L2, Z, PPGB), receptors (EGFR, lamin B, poliovirus), solute/ion carrier transporters, and MHC molecules (HLA-C, HLA-A, GRP58).

In set 14 (Figure 3B), genes whose expression peaked at the G_1_/S phase transition were observed, though a greater number of genes relative to set 12 with similar expression patterns and functions were found. For example, we observed up-regulation of apoptosis regulators (UDP-galactose ceramide glucosyltransferase, BAX, BAX inhibitor 1, TRAIL receptor 2, thioredoxin peroxidase, CD47, API5-like 1), receptors/adhesion proteins (CCRL2, LIFR, EGFR, FGFR1, syndecan 4, syndecan 1, IL-4R, IL-13R, lymphotoxin B receptor), signaling mediators (Grb2, AKAP1, IRAK1, CaM-kinase II, calcineurin), and proteins involved in transcriptional regulation (BAF60C, NFI/C, ATF6). Interestingly, 26 genes in this cluster were related to the ER-Golgi protein transport pathway, suggesting a dependence on efficient protein processing and intracellular transport. These findings suggest an increase in Tat-induced receptor-mediated signaling and transcription, and most importantly, the increased expression of membrane proteins and antigens involved in promoting HIV-1 replication and immune evasion.

On the other hand, set 5 (Figure 3C) shows 20 genes whose expressions peaked at late G_1_, early S, and then again at G_2_, while their expressions were lowest at early G_1_. This set contains primarily ribosomal subunit genes. We previously observed very similar results in our microarray experiment using Tat-expressing H9 cells [5], where we saw a significant up-regulation of numerous ribosomal subunit genes and translation initiation factors. The dramatic temporal expression of the ribosomal subunits for the 40S and 60S components in early S, as seen in set 5, may be indicative of a critical coupling of transcription and translation for efficient viral RNA production.

### Tat-mediated gene expression during G_1_/S phase

Using a complementary technique for unsupervised clustering, we looked at those genes that were induced by HIV-1 Tat during the late G_1 _phase and the G_1_/S phase transition since our previous findings indicated that these cell cycle phases were starting points for transcription of the HIV-1 long terminal repeat (LTR) and activated viral transcription [2]. The SOM analysis makes it easier to visualize the dramatic cell cycle effects of Tat on the total gene dataset. In this analysis, red areas indicate up-regulated genes, while blue indicates down-regulated genes, and yellow represents minor effects on gene expression. The U-matrix allows visualization of those clusters in the SOM that show significant expression changes. Each hexagon or neuron corresponds to a group of genes with similar expression patterns. We performed 3 filters to generate SOMs, with the last filter being the most restrictive (Figure 4). The most restrictive list includes genes that show a 3-fold increase or decrease in expression between the experimental and control samples at each time point. For this particular SOM, genes were removed if their average signal ratio fell between 0.333 and 3.0 across all time points tested and displayed absent calls at any time point.

Using the SOM analysis from the third filter (Figure 4), we observed a similar transcription profile throughout the G_1 _phase, with a marked difference at the G_1_/S transition. This is seen with the dramatic induction of those genes represented in the red and dark red neurons at the bottom right portion of the G_1_/S SOM. Repression of genes on the left side of the G_1 _component plane, when cells enter the G_1_/S transition, was also observed. Interestingly, the G_1_/S profile remained relatively constant through the S phase, while upon entering G_2_, there was an overall reduction in Tat-mediated gene activation. This can be seen with the greater percentage of blue neurons at the G_2 _phase concomitant with a reduction of dark red neurons. We generated a list of genes up-regulated at the G_1_/S transition that were seen in both k-means and SOM clustering analyses (Table 1). Bolded genes are those that have already been shown to be involved in HIV-1 infection. It is important to note that there were a significant number of genes that were identified as similarly dysregulated by using both the k-means and SOM analyses across all time points.

**Table 1:** SOM and K-means Analysis of Tat-upregulated genes at the G_1_/S phase.^a^

**Gene Ontology**	**Accession #**	**Gene Title**	**Gene Symbol**	**Unigene ID**
Transcription/	D83782	SREBP cleavage-activating protein	SCAP	Hs.437096
DNA binding	AC004770	fatty acid desaturase 3	FADS3	Hs.21765
Enzymes	Y08685	serine palmitoyltransferase, long chain base subunit 1	SPTLC1	Hs.90458
	D50840	UDP-glucose ceramide glucosyltransferase	UGCG	Hs.432605
	AF038961	mannose-P-dolichol utilization defect 1	MPDU1	Hs.95582
	U67368	exostoses (multiple) 2	EXT2	Hs.75334
	M22488	bone morphogenetic protein 1	BMP1	Hs.1274
	AF002668	degenerative spermatocyte homolog, lipid desaturase (Drosophila)	DEGS	Hs.299878
	AB016247	sterol-C5-desaturase-like	SC5DL	Hs.287749
	**X15525**	**acid phosphatase 2, lysosomal**	**ACP2**	**Hs.75589**
	D13643	24-dehydrocholesterol reductase	DHCR24	Hs.75616
	AF020543	palmitoyl-protein thioesterase 2	PPT2	Hs.332138
	AL050118	fatty acid desaturase 2	FADS2	Hs.388164
	**M16424**	**beta-hexosaminidase A (alpha polypeptide)**	**HEXA**	**Hs.411157**
	L13972	sialyltransferase 4A (beta-galactoside alpha-2,3-sialyltransferase)	SIAT4A	Hs.356036

Membrane/	**D79206**	**syndecan 4 (amphiglycan, ryudocan)**	**SDC4**	**Hs.252189**
Antigens	**M90683**	**HLA-G histocompatibility antigen, class I, G**	**HLA-G**	**Hs.512152**
	**X58536**	**major histocompatibility complex, class I, C & B**	**HLA-C, B**	**Hs.77961**
	AF068227	ceroid-lipofuscinosis, neuronal 5	CLN5	Hs.30213
	U72515	putative protein similar to nessy (Drosophila)	C3F	Hs.530552
	X85116	stomatin	STOM	Hs.439776
	Z26317	desmoglein 2	DSG2	Hs.412597
	**S90469**	**P450 (cytochrome) oxidoreductase**	**POR**	**Hs.354056**

Receptors/Ligands	U97519	podocalyxin-like	PODXL	Hs.16426
	AI263885	interleukin 27 receptor, alpha	IL27RA	Hs.132781
	**U60805**	**oncostatin M receptor**	**OSMR**	**Hs.238648**
	M63959	low density lipoprotein receptor-related protein associated protein 1	LRPAP1	Hs.75140
	L25931	lamin B receptor	LBR	Hs.435166
	**X00588**	**epidermal growth factor receptor**	**EGFR**	**Hs.77432**
	**M25915**	**clusterin**	**CLU**	**Hs.436657**
	**X87949**	**heat shock 70 kDa protein 5 (glucose-regulated protein, 78 kDa)**	**HSPA5**	**Hs.310769**

Proteases	AF032906	cathepsin Z	CTSZ	Hs.252549
	AB001928	cathepsin L2	CTSL2	Hs.87417
	**Y00264**	**Amyloid beta (A4) precursor protein**	**APP**	**Hs.177486**

Protein transport/Chaperone	D83174	serine (or cysteine) proteinase inhibitor, clade H, member 1	SERPINH1	Hs.241579
	Z49835	glucose regulated protein, 58 kDa	GRP58	Hs.110029
	X97335	A kinase (PRKA) anchor protein 1	AKAP1	Hs.78921
	X90872	gp25L2 protein	HSGP25L2G	Hs.279929
	D49489	thioredoxin domain containing 7 (protein disulfide isomerase)	TXNDC7	Hs.212102
	AF013759	calumenin	CALU	Hs.7753
	AL008726	protective protein for beta-galactosidase (galactosialidosis)	PPGB	Hs.118126
	Z50022	pituitary tumor-transforming 1 interacting protein	PTTG1IP	Hs.369026
	**AA487755**	**FK506 binding protein 9, 63 kDa**	**FKBP9**	**Hs.497972**

Ion channel/transporter	U81800	solute carrier family 16, member 3	SLC16A3	Hs.386678
	M23114	ATPase, Ca++ transporting, cardiac muscle, slow twitch 2	ATP2A2	Hs.374535
	J04027	ATPase, Ca++ transporting, plasma membrane 1	ATP2B1	Hs.20952
	AL049929	ATPase, H+ transporting, lysosomal accessory protein 2	ATP6AP2	Hs.183434
	AL096737	solute carrier family 5, member 6	SLC5A6	Hs.435735

Unknown/Other	AF052159	protein tyrosine phosphatase-like, member b	PTPLB	Hs.5957
	D14658	KIAA0102 gene product	KIAA0102	Hs.87095
	AI867349	nicastrin-like protein	NICALIN	Hs.24983
	AL031228	solute carrier family 39 (zinc transporter), member 7	SLC39A7	Hs.66776
	X57398	nodal modulator 1, 2, 3	NOMO1, 2, 3	Hs.429975

^a ^Bolded genes indicate those genes upregulated at the G1/S transition (found using both SOM and k-means analyses)

Numerous signaling receptors were shown to be up-regulated upon Tat expression. The oncostatin M receptor is normally bound by the IL-6 cytokine family member and is increased in HIV-1 infection [11]. Interestingly, oncostatin M has been shown to stimulate the production of immature and mature T cells in the lymph nodes of transgenic mice [12]. It has also been shown that cdk9, a component of pTEFb, can also bind gp130, which is a common subunit recognized by the IL-6 cytokine family [13]. Expression of the 4-1BBL cytokine, a T-cell co-stimulatory molecule (i.e. induces IL-2 production and T-cell proliferation) that is involved in the antigen presentation process and generation of a CTL response was also increased [14,15].

Similarly, we observed the up-regulation of LFA-3, ICAM-1, and other membrane proteins and receptors. These membrane proteins serve as additional activation signals and molecules involved in the transmission of free virus to bystander, uninfected cells [16-18]. Interestingly, a recent report illustrates the ability of soluble ICAM (sICAM) to promote infection of resting cells and cell cycle progression after initiating B and T cell interactions [19]. Syndecan 4 was also up-regulated by Tat at the G_1_/S phase. Syndecans are a type of heparan sulfate proteoglycan (HSPG) that is able to efficiently attach to HIV-1 virions, protect them from the extracellular environment, and efficiently transmit the captured virions to permissive cells [20]. We also observed the up-regulation of the CXCR4 co-receptor that is critical for infection by X4 HIV-1 strains. Likewise, the SDF receptor 1 had increased expression. SDF-1 is the ligand for the CXCR4 co-receptor and can block HIV-1 infection via co-receptor binding. Therefore, the expression of the SDF receptor 1 could serve as an alternate binding site for SDF-1, allowing CXCR4 to be available for HIV-1 gp120/gp41-binding. Fractalkine, the ligand for the CX3CR1 receptor, has been shown to be important in the adhesion, chemoattraction, and activation of leukocytes [21], was also up-regulated by Tat expression. Overall, these proteins serve to increase the efficiency of HIV-1 infection, transmission to other cells, activation of T cells, and the recruitment of circulating leukocytes to infection sites.

A critical feature of HIV-1 infection is its ability to evade host immune responses and subsequently create a state of immunodeficiency. Previous studies have shown the ability of HIV-1 Nef to decrease the expression of CD4, HLA-A, and HLA-B, while having no effect on HLA-C or HLA-D, which allows for host cell survival and permits productive viral progeny formation prior to immune recognition and eventual apoptosis [22,23]. HLA-A and HLA-B allow for efficient CD8^+ ^cytotoxic T lymphocyte (CTL) detection. Since it has been demonstrated that HLA-C and HLA-E are needed for protection from natural killer (NK) cell-mediated death [23], the up-regulation of HLA-C by Tat suggests similar host cell survival-directed functions for both Tat and Nef. Interestingly, HLA-G has been shown to be up-regulated in both monocytes and T lymphocytes of seropositive individuals, though its relation to infection and pathogenesis remains to be determined [24].

Collectively, SOM and k-means analyses catalog a set of genes representative of a close interplay between promoting and inhibiting factors induced by Tat. These findings, coupled with the up-regulation of signaling receptors involved in cell growth and survival, illustrate an intrinsic ability of HIV-1 Tat in regulating immune evasion, viral transmission, cell cycle progression and subsequent apoptosis. Importantly, these results delineate a variety of cellular gene products, both previously characterized with respect to HIV-1 and those uncharacterized, to be directly or indirectly induced by Tat expression. A plausible notion is that during activated transcription, HIV-1 hijacks the host cell machineries to promote its own replication, while concurrently directing a certain minimal level of cell survival until the virus reaches its critical point of progeny formation and subsequent virus-induced cell cycle block and apoptosis at the G_2 _phase.

### siRNA-mediated validation of cellular HIV-1 therapeutic targets

Using siRNAs targeted at several Tat-induced host cellular gene products, we examined the significance of our synchronized microarray data on a few genes we thought were critical for productive viral progeny formation. Based on the 32 arrays (16 eTat and 16 pCep4) in this study, we generated a list of Tat-induced genes that included those genes displaying two or more present calls on the eTat chips (present on at least 2 of 16 chips) while having 16 absent calls in the control pCep4 chips. We hypothesized that genes which were consistently (at various cell cycle phases) induced/repressed by Tat and were absent from the control pCep4 chips, would be the most important and specific for the Tat-mediated effects on the viral life cycle or host cell cycle progression. We also identified genes that displayed at least four and at least eight present calls across all 16 eTat chips and displayed all absent calls across all 16 pCep4 chips [see Additional File 4 and Methods]. Finally, the two present call gene list was screened against the Hu95 microarray data indexed at the Children's National Medical Center (CNMC) in Washington, D.C. This analysis was executed to identify those genes only induced by Tat, while never induced in a myriad of other human genetic diseases and tissues whose data is hosted at CNMC. Those genes that were 100% absent or 50.1% to 99.9% absent across all the Hu95 data in the database were compiled and listed (Table 2). This list of genes has potential to be very specific cellular therapeutic targets.

**Table 2:** Tat-upregulated genes not induced in other genetic diseases profiled.

**Accession #**	**Fold Change**	**Gene Name**
D13243	1.9	Pyruvate kinase L
Z49194	4.1	Pou2AF1 (OBF-1)
AF072099	3.1	LILRB4
U61836	0.2	SMOX
J00117	10.8	CGB
X02612	2.2	Cytochrome P(1)-450 (CYP1A1)
Y12851	0.8	P2X7 receptor
AI349593	0.6	Similar to hemoglobin epsilon chain
AF055007	3.9	MARCH-III
AB002449	3.9	Hypothetical gene
AA203545	1.9	Unknown

Based on a literature search of our initial list of dysregulated genes (from the K-means, SOMs, and present call gene list analyses) and from the CNMC screen, we have a comprehensive list of potential targets. Through the exhaustive literature search, we looked for genes that were previously characterized as necessary for HIV-1 replication and/or progeny formation and identified HIV-1 Rev binding protein 2, Pou2AF1 (OBF-1), cyclin A1, PPGB, EXT2, and HEXA for further analysis. The HIV-1 Rev binding protein 2 has been characterized as having high homology to the *S. cerevisiae *Krr1p protein, which is a nucleolar protein, and has been shown to be critical for 18S rRNA synthesis and subsequent 40S ribosome synthesis and cell viability [25-27]. Therefore, ablation of the HIV-1 Rev binding protein 2 should theoretically inhibit virus replication and possibly direct infected cells towards apoptosis. The HIV-1 LTR contains four potential binding sites for the Oct-1 transcription factor and Oct-1 has been shown to interact with Tat [28]. OBF-1 interacts with Oct-1 and Oct-2, acting as a B lymphocyte-specific transcriptional coactivator of B cell activation and maturation, as well as induction of immunoglobulins. It is also activated in T cells upon TCR signaling [29]. Recently, OBF-1 was found to up-regulate CCR5 co-receptor surface expression and fusion to the Env protein of R5 strains, the predominant strain found during initial infection [29]. Therefore, we predict that this factor is repressed upon the onset of AIDS, which is usually correlated with a R5 to X4 HIV-1 strain switch. Cyclin A1, which binds and regulates cdk2 and cdk1, was also chosen for targeted inhibition since it is important during the S and G_2 _phases of the cell cycle, both of which are important for the viral life cycle [5,30]. Cyclin A1 has also been shown to bind Rb family members, the p21/waf1 family of endogenous cdk inhibitors, as well as the E2F-1 transcription factor, all of which are important in the regulation of cell cycle progression and HIV-1 progeny formation [4,6,31-34].

Based on the importance of viral attachment, entry, and membrane fusion in the course of infection, we also chose to inhibit expression of the PPGB protein, which forms a heterotrimeric complex with the lysosomal enzymes β-galactosidase and neuraminidase (NA). Though there have been no reports on the contribution of PPGB in HIV-1 infection, a number of reports have illustrated the importance of NA in increasing the efficiency of viral binding and entry [35,36]. NA is a sialidase that exposes sites on the HIV-1 gp120 surface protein, enabling greater interaction between gp120 and the CD4/co-receptor complex, which consequently increases syncytium formation and single-round infection by both X4 and R5 HIV-1 isolates. These findings coupled with the importance of HSPGs, illustrate the importance of membrane proteins and their modifications on both viral attachment and entry processes. Cellular proteins involved in the fusion and entry processes of infection may play a greater role in extracellular Tat-mediated effects, such as bystander cell infection.

The EXT2 and HEXA gene products were also targeted since they displayed present calls in at least half of the eTat chips and showed no induction in the pCep4 chips [see Additional File 4]. EXT2 is a putative tumor suppressor with glycosyltransferase activity that is involved in the chain elongation step of heparan sulfate biosynthesis [37]. HEXA is involved in ganglioside GM2 degradation and is a member of a subfamily of glycosyl hydrolases [38]. It has been established that GM2 levels are significantly increased in HIV-1 infection, as is seen both *in vitro *and *in vivo *from seropositive individuals [39,40]. Surprisingly, both groups showed that anti-GM2 IgM antibodies caused complement-mediated cytolysis of infected cells. We propose that inhibiting HEXA would increase the levels of circulating GM2 *in vivo*, thereby creating a more pronounced level of infected cell cytolysis.

**Figure 5:**
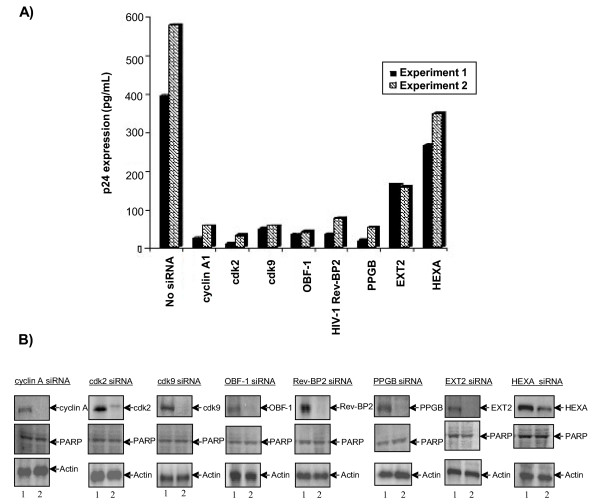
**Representative siRNA-directed inhibition of HIV-1 replication. **(A) Using two candidate siRNAs per gene shown, each siRNA was transfected into HIV-1 latently infected OM-10.1 cells at mid log phase of growth. Following transfection, viral activation, and treatment, supernatants were collected and analyzed for p24 Gag expression by ELISA. The white crossed bars represent the first set of experiments, while the black bars represent the second run performed in an identical manner. (B) For Western blots, protein samples (one hundred micrograms of each extract) were separated on SDS-PAGE and then transferred to an Immobilon-P (polyvinylidene difluoride; Millipore) membrane and blocked with 5% fat-free milk (in TNE50/0.1% Nonidet P-40). Membranes were incubated overnight with various primary antibodies, and reactive complexes were developed with protein G-labeled ^125^I and visualized with a PhosphorImager scanner (Amersham Biosciences).

Using HIV-1 latently infected OM 10.1 T cells, which contain a single copy of silent full length wild type infectious provirus, we transfected 10 μg of each siRNA (2 for each representative gene) into cells. After 48 hrs, TNF-α was added for 2 hours to induce the latent virus and normal cell cycle progression. Samples were collected at 72 hrs post-TNF-α treatment and subjected to p24 Gag ELISA and western blot analysis. Cells that were not transfected with any siRNA were used as the negative control sample, while cdk2 and cdk9-targeted siRNAs served as positive controls. As seen in Figure 5A, the majority of siRNAs demonstrated some efficacy in inhibiting p24 expression. Ablation of EXT2 had a moderate effect (~2 fold reduction), while the HEXA siRNA had a negligible effect (<1 fold reduction). While the cdk2- and cdk9-mediated inhibition of HIV-1 replication was expected [41,42], the potency of the other siRNAs were very dramatic. Interestingly, the most effective siRNAs were involved in cell cycle progression and/or transcription (cdk2, cdk9, cyclin A1, and OBF-1), RNA pathways (HIV-1 Rev binding protein 2), or membrane protein modification (PPGB). While EXT2 has been shown to be important in heparan sulfate synthesis, HSPGs are most important for cells that do not express large amounts of CD4, such as macrophages [20]. Thus, EXT2 degradation should only affect infection and replication in cells devoid of CD4.

**Figure 6:**
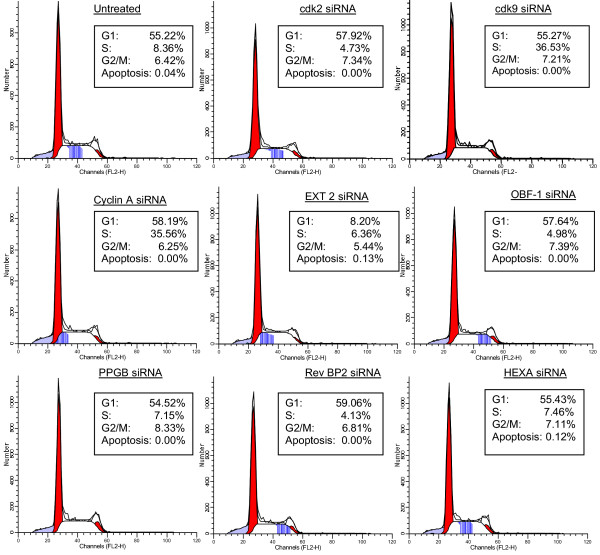
**FACS analysis of PI stained OM10.1 cells**. The stained cells were analyzed for red fluorescence (FL2) on a FACScan (Becton Dickinson, San Jose, CA), and cell distribution in the G_1_, S, and G_2_/M phases of the cell cycle was calculated from the resulting DNA histogram with Cell FIT software, based on a rectangular S-phase model. A sub-G1 population was considered as an apoptotic population.

We also performed series of western blots to measure the efficiency of inhibition from each of siRNAs tested. As shown in Figure 5B most siRNA treatments dropped the protein level by more than 90%, except for the HEXA gene. None of siRNAs inhibited actin gene expression or PARP degradation (an indicator of active apoptosis), implying that the siRNA targets were not toxic in these transient experiments. We finally performed simple FACS analysis using PI staining and saw no apparent cell cycle or apoptotic effects (Figure 6). Although, we have never been able to inhibit HEXA translation completely in OM10.1 cells (or three other infected cell lines), data on HEXA indicates that even a 50% drop in protein levels maybe sufficient to increase GM2 levels, thereby increasing a more pronounced rate of viral production.

**Figure 7:**
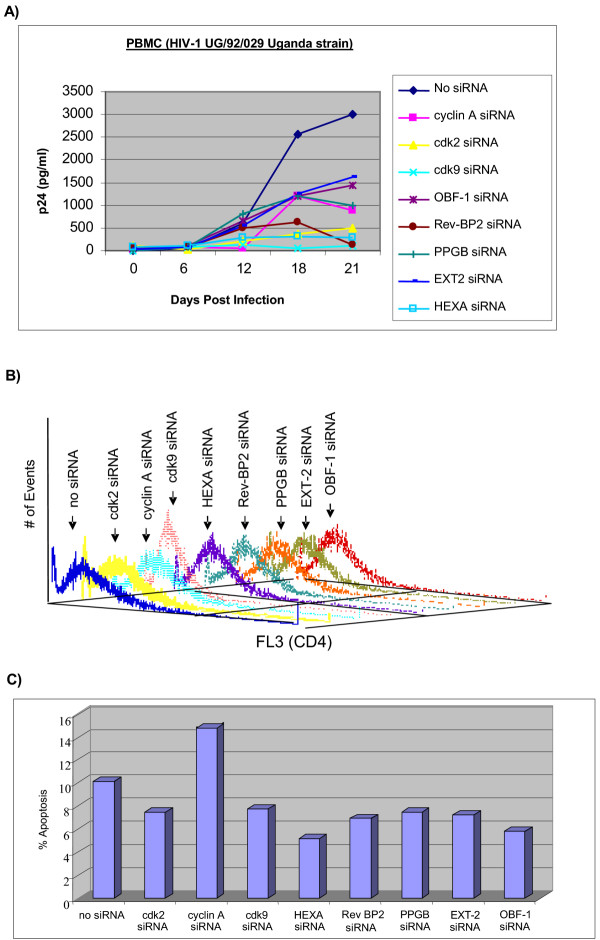
**Effect of representative siRNA treatment in PBMC field isolate HIV-1 infection. **Approximately 5 × 10^6 ^Phytohemagglutinin-activated PBMC were kept in culture for two days prior to infection. PBMC were first treated for 48 hrs with 10 μg of the various siRNAs and then infected with SI (UG/92/029 Uganda strain, subtype A envelope, 5 ng of p24 gag antigen) strain of HIV-1 obtained from the National Institutes of Health (NIH) AIDS Research and Reference Reagent Program. After 8 h of infection, cells were washed and fresh media was added. Samples were collected every sixth day and stored at -20°C for p24 gag enzyme-linked immunosorbent assay (ELISA). Media from infected cell lines was centrifuged to pellet the cells and supernatants were collected and diluted to 1:100 to 1:1,000 in RPMI 1640 prior to analysis. Supernatants from the infected PBMC were collected and used directly for the p24 antigen assay. The p24 gag antigen level was analyzed using the HIVAG-1 Monoclonal Antibody Kit (Abbott Laboratories, Diagnostics Division). (B) PBMCs stimulated with PHA were treated with appropriate siRNA prior to HIV infection and stained for presence of surface CD4 on activated cells. Prior to infection, 1/5 of the samples were processed for CD4 and PI staining. Cells were then collected and washed twice with PBS containing FCS and NaN_3_. Cells were stained on ice for with human tri-color-labeled anti-CD4 (Catalog Laboratories) at a 1:10 dilution. Stained cells were next washed two times in PBS containing FCS and NaN_3 _and fixed in paraformaldehyde followed by analysis by FACS. (C) FACS analysis of PI stained cells from panel B. Sub-G1 population was scored as apoptotic population in each siRNA treated cell.

Next, we performed a similar set of experiments in PBMCs infected with a HIV-1 field isolate and treatment with various siRNAs. Activated PBMCs were first treated with 10 μg of each siRNA for 48 hours and subsequently infected with a field HIV-1 isolate (UG/92/029 Uganda strain, sub-type A envelope). Supernatants were collected every six days for Gag p24 assay. Results in Figure 7A indicate that siRNA's against cdk9, cdk2, HEXA, and Rev-BP2 were the most potent inhibitors, followed by siRNAs against cyclin A, OBF-1 and PPGB, and the least amount of inhibition with EXT-2 siRNA. Control experiments using antibody staining against CD4 on activated PBMCs treated with each siRNA for 48 hours prior to HIV-1 infection showed no appreciable differences, except a minor drop with cdk2 siRNA (~5%) in CD4 levels (Figure 7B), and a PI staining of the same cells also showed no significant apoptosis except for a minor drop with cyclin A siRNA (~5%, Figure 7C), implying that the siRNA treatment in general did not significantly alter the expression of CD4 levels prior to viral infection. Collectively, these results are somewhat similar to the latent OM10.1 treatments and imply that these genes could be a potential target in both cell lines and primary infections.

**Figure 8:**
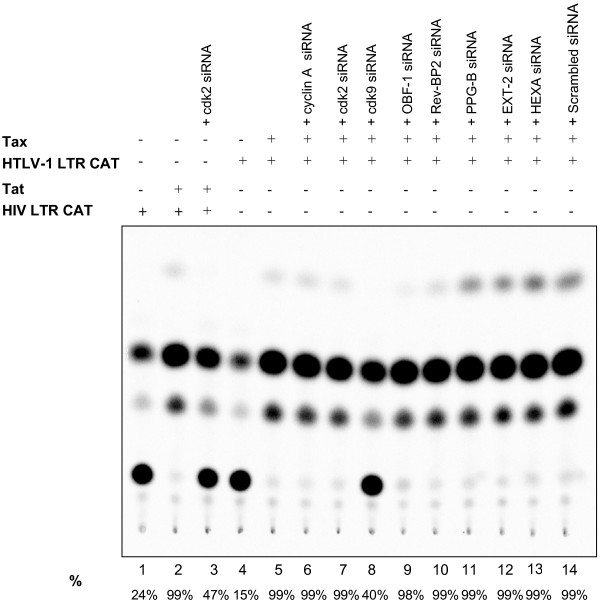
**CAT assays with HIV-LTR-CAT and its activator Tat, and HTLV-LTR-CAT and its positive activator Tax. **Lymphocyte (CEM, 12D7) cells were grown to mid log phase and were processed for electroporation according to a procedure published previously [52]. The cells were washed with phosphate-buffered saline and resuspended in RPMI 1640. They were next transfected with reporter constructs (HIV-LTR-CAT or HTLV-LTR-CAT; 3 ug of each), their respective activators (Tat or Tax; 4 ug each) or with various siRNAs (10 ug each). Lanes 1–3 serve as positive controls for basal, activated transcription and effect of cdk2 siRNA on inhibition of HIV-1 LTR. Lanes 4–14 are basal, activated transcription and effect of various siRNAs on HTLV- LTR-CAT. Only cdk9 siRNA showed an appreciable amount of suppression on Tax activated HTLV-LTR (lane 8). CAT % conversations are listed below the diagram.

Finally, we asked whether the identified gene lists from our siRNA experiments were specific to HIV-1 transcription or could they also inhibit other viral activated transcriptions. We therefore performed CAT assays with either HIV-LTR-CAT and its activator Tat (as positive controls, Figure 8, Lanes 1–3) or HTLV-LTR-CAT and its positive activator Tax (Figure 8, lanes 4–14). Results in Figure 8 show that HIV-1 activated Tat can be suppressed with cdk2, however none of the siRNA treatments inhibited HTLV-1 Tax activated transcription except cdk9 siRNA. This result is somewhat expected since cdk9 is known to be involved in general transcription elongation, and is consistent with a recent report indicating that Tax might have a role in transcription elongation [43,44].

## Conclusion

### Potential therapeutic targets of HIV-1 Tat-induced cellular genes

We believe that our current results are by no means the ultimate list of genes altered by HIV-1 Tat. Some of the limitations of our experiments include: constant presence of Tat in cells as compared to possible transient expression of Tat in HIV-1 infected cells, possible indirect effect of Tat on gene expression, and lack of using various Tat clades (i.e., from clades B, E, and C), which may have a different rate and set of activated genes *in vivo*. However, we believe the current study is an ongoing attempt to narrow down which cellular genes are critical in Tat regulation and therefore define a minimal set of potential targets for therapy.

Based on exhaustive and stringent data analysis, we have compiled a list of gene products that may serve as potential therapeutic targets for the inhibition of HIV-1 replication (Table 1 and 2). Table 1 specifies Tat-induced cellular genes at the G_1_/S transition, while Table 2 lists those genes that were observed to be up-regulated by Tat while displaying no induction in the myriad of genetic diseases and diverse tissues and cell types screened at CNMC. As observed in both tables and the initial screening of genes displaying at least two present calls, several genes have been established as important for HIV-1 infection and replication, including OBF-1 [29,45], complement factor H related 3 [46], CD4 receptor, ICAM-1 [18], NA [35,36], and cyclin A1 [8,47].

There were also several genes that have not been published in relation to HIV-1 infection and may also be novel and efficacious therapeutics. These include FGFR and EGFR, the latter of which has been targeted against various cancers and inhibits cancer-associated angiogenesis and subsequent metastasis [48]. Concerning HIV-1 infection and replication, some potentially important proteins that have not been previously characterized with respect to HIV-1 and thus necessitate further study, seem to be the CAP-binding protein complex interacting protein, tropomyosin 2 beta, BTG3, the IL-10R, PPGB, and cathepsins Z and L2 [see Additional File 4 and Tables 1 &2]. Though not established, the CAP-binding protein complex is most likely involved in translation processes. Tropomyosin 2 beta was found to interact with FRP1, which is important in the regulation of HIV-1 virus-mediated cell fusion and possibly syncytium formation [49]. Also, therapeutics against individual gene products or a cocktail containing inhibitors for ICAM-1, LFA-3, DC-SIGN, all syndecan isoforms, PPGB, clusterin and other adhesion/membrane proteins important in viral transmission may, alone or in combination with Fuzeon/T20, significantly abrogate the infection of circulating lymphocytes and other cells that are able to support viral infection and replication.

Recently a report by Krishnan and Zeichner described experiments associated with changes in cellular gene expression that accompany the reactivation of the lytic viral cycle in cell lines chronically infected with HIV-1. They found that several genes exhibited altered expression in the chronically infected cells compared to the uninfected parental cells prior to induction into lytic replication including genes encoding proteasomes, histone deacetylases, and many transcription factors [50]. Although it is difficult for us to compare our results with Krishnan and Zeichner due to difference in cell types, presence of all HIV-1 ORFs as compared to our study where there was only Tat present, and the difference in cell cycle stages, however, we did a general comparison and found some overlap between our list of dysregulated genes and theirs – this overlap includes genes coding for splicing factors, proteasomes, and heat shock proteins. We compared our SOM and k-means analyses (Table 1) from which we found genes that displayed differential expression at the G1/S phase and found three intersecting genes as well as some genes that are very closely related to genes listed in the Krishnan table (e.g. genes coding for a different subunit of a protein); these genes are listed in Table 3. The first part of Table 3 contains three genes that fell in both our SOM and k-means analyses and the Krishnan table (bold genes) and the genes from our SOM and k-means analyses that are closely related to genes in the Krishnan table. Collectively, the list of common genes indicates the involvement of HIV-1 Tat in splicing, transport of RNA, an acceleration of cell cycle stages. All of these genes fall into pathways that have previously been reported to be regulated by Tat, including stabilization of critical transcription units (i.e., Hsp70 stabilization of Cdk9/cyclin T1 complex), splicing and nuclear transport (i.e., the SR protein ASF/SF2; Tat-SF1), translation (5'-terminal TAR recognition by eukaryotic translation initiation factor 2), and degradation of critical factors needed for cell cycle progression using the proteosome pathway (i.e., analogous to HPV E6 binding to p53 and its degradation resulting in loss of check point, ubiquitin/proteasome degradation of IkappaB(alpha) and release of active NFkB, or CD4 glycoprotein degradation through the ubiquitin/proteasome pathway). Therefore these results imply that Tat regulates these apparently discrete pathways, at least in case of pre-mRNA processing, where transcription initiation/early elongation complex directly controls every aspect of subsequent pre-mRNA processing including capping at the 5' end, intron recognition and removal by splicing, the 3' end cleavage and polyadenylation, and release of the mature mRNA from the site of transcription and export to the cytoplasm for translation [51].

**Table 3:** A set of common genes regulated by Tat in both Tat expressing cells and HIV-1 infected cells.

**Probe Set ID**	**Accession #**	**Gene Description**
**34083_at**	**AA311181**	**splicing factor, arginine/serine-rich 9**
**35323_at**	**U78525**	**eukaryotic translation initiation factor 3, subunit 9 (eta, 116 kD)**
**31858_at**	**X07315**	**nuclear transport factor 2**
32165_at	L41887	splicing factor, arginine/serine-rich 7 (35 kD)
32556_at	X64044	U2 (RNU2) small nuclear RNA auxiliary factor 2
33372_at	AI189226	RAB31, member RAS oncogene family
39628_at	AI671547	RAB9A, member RAS oncogene family
2029_at	N36267	Rho GTPase activating protein 5
35255_at	AF098799	RAN binding protein 7
1191_s_at	AB003102	proteasome (prosome, macropain) 26S subunit, non-ATPase, 11
1192_at	AB003103	proteasome (prosome, macropain) 26S subunit, non-ATPase, 12
37350_at	AL031177	proteasome (prosome, macropain) 26S subunit, non-ATPase, 10
1104_s_at	M11717	heat shock 70 kD protein 1A
36614_at	X87949	heat shock 70 kD protein 5 (glucose-regulated protein, 78 kD)
35467_g_at	W73046	DnaJ (Hsp40) homolog, subfamily B, member 12

While some of these proteins have available inhibitors, the majority of the potential cellular targets for HIV-1 therapeutics do not have known specific inhibitors. Thus, much effort must be allocated for the elucidation and design of specific inhibitors, concurrent with the growing plausibility of siRNA-based therapeutics. Another important factor in designing inhibitors for cellular targets, as shown with potential cancer therapeutics, is the necessity to target cellular gene products with redundant functions. If a certain cellular protein kinase, receptor, membrane protein, or cytokine/chemokine is inhibited, it may have adverse effects that make the drug impractical for clinical trials and use. However, the presence of two or more proteins with similar functions, with only one being critical for HIV-1 and thus targeted, may allow for the decreased possibility of side effects. This is especially true for targeting redundant molecules (i.e., cdk2), where they are nonessential during mammalian development and are likely replaced by other kinases. Similarly, once specific inhibitors are elucidated, a major resulting challenge is generating a combinatorial therapeutic regimen that is effective in sub-lethal doses (submicromolar or nanomolar range).

## Methods

### Cell culture

HeLa CD4^+ ^cells containing either an epitope-tagged (the influenza epitope at the C terminus of Tat 1–86) eTat plasmid or the parental control vector pCep4 were used [2]. All cells were cultured in RPMI 1640 containing 10% fetal bovine serum, 1% streptomycin/penicillin, and 1% L-glutamine (Quality Biological) at 37°C in 5% CO_2_.

### Cytoplasmic RNA isolation

Cells were centrifuged at 4°C, 3000 rpm for 10 min., quickly washed with D-PBS without Ca^2+^/Mg^2+^, and centrifuged twice. Pelleted cells were immediately frozen at -80°C until all time points were collected. Cytoplasmic RNA was isolated utilizing the RNeasy Mini Kit (Qiagen, Valencia, CA) according to manufacturer's directions with the addition of 1 mM dithiothreitol in Buffer RLN. Isolated RNA was quantitated by UV spectrophotometric analysis and 3 μg of RNA was visualized on a non-denaturing 1% agarose TAE gel for quality and quantity control.

### Lymphocyte Transfection

Lymphocyte (CEM, 12D7) cells were grown to mid log phase and were processed for electroporation according to a procedure published previously [52]. The cells were centrifuged and then washed with phosphate-buffered saline without Mg2+ or Ca2+ twice and resuspended in RPMI 1640 at 4 × 10^5 ^cell/0.25 ml. The CEM cells (0.25 ml) were transfected with the plasmid DNAs of HIV-LTR-CAT or HTLV-LTR-CAT (3 ug of each) either alone or in combination with Tat or Tax (4ug each). 10 μg of the various siRNAs were also mixed in with reporter and/or appropriate transactivator prior to electroporation. The mixture of cells, plasmid DNAs, and siRNAs were then transferred to a cuvette and electroporated with fast charge rate, at 230 V, and capacitance of 800 microfarads. Cells were then plated in 10 ml of complete RPMI 1640 medium for 18 h prior to harvest and CAT assay. For CAT assays, standard reaction was performed by adding the cofactor coenzyme A to a microcentrifuge tube containing cell extract and radiolabeled chloramphenicol, in a final volume of 50 μl and incubated at 37°C for 1 h. The reaction mixture was then extracted with ethyl acetate. It was then separated by TLC on silica gel plates (Baker-flex silica gel TLC plates) using the chloroform:methanol (19:1) solvent system. The resolved reaction products were then detected by exposing the plate to a PhosphorImager cassette.

### Immunoprecipitation/Western Blot Analysis

Immunoprecipitations of tat protein were performed as described previously [2]. Cellular protein (100 μg) was mixed with monoclonal 12CA5 antibody (2.5 μg) for 2 h at 4°C. Protein A + G agarose beads (5 μl; Calbiochem, Inc.) were added and incubated at 4°C for another 2 h. The immunoprecipitated complex was then spun down and washed with buffer D containing 500 mM KCl (three times; 1 ml each). Samples were eluted with HA- peptide for 4 hrs at 37 C on a rotator, and eluted complexes were separated on a 4–20% SDS-polyacrylamide gel electrophoresis gel, and Western blot analysis was performed with anti-Tat monoclonal antibody. Antigen/antibody complexes were detected with ^125^I Protein G.

### CD4 staining of human cells

Human PBMCs stimulated with PHA were treated with appropriate siRNA prior to HIV infection. Activated PBMCs were first treated with 10 μg of each siRNA for 48 hours and subsequently infected with a field HIV-1 isolate (UG/92/029 Uganda strain, subtype A envelope, 5 ng of p24 gag antigen) [53]. Prior to infection, 1/5 of the samples were processed for CD4 and PI staining. Cells were then collected and washed twice with PBS containing 5% FCS and 0.05% NaN_3_. Cells were stained on ice for 30 minutes with human tri-color-labeled anti-CD4 (Catalog Laboratories) at a 1:10 dilution. Stained cells were next washed two times in PBS containing 5% FCS and 0.05% NaN_3 _and fixed in 1% paraformaldehyde followed by analysis by FACS.

### Cell cycle analysis

The eTat and pCep4 cells were either blocked with hydroxyurea (G_1_/S blocker, 2 mM) or nocodazole (G_2_/M blocker, 50 ng/ml). Cells were washed with PBS and released with complete medium. Samples were collected every 3 hrs and cytoplasmic RNA was isolated. Single-color flow cytometric analysis of DNA content (PI staining) was performed on both cell types [2]. Stained cells (including OM10.1) were analyzed for red fluorescence (FL2) on a FACScan (Becton Dickinson, San Jose, CA), and cell distribution in the G_1_, S, and G_2_/M phases of the cell cycle was calculated from the resulting DNA histogram with Cell FIT software, based on a rectangular S-phase model.

### PBMC infection

Phytohemagglutinin-activated PBMC were kept in culture for two days prior to each infection. Isolation and treatment of PBMC were performed by following the guidelines of the Centers for Disease Control. Approximately 5 × 10^6 ^PBMC were first treated for 48 hrs with 10 μg of the various siRNAs and then infected with SI (UG/92/029 Uganda strain, subtype A envelope, 5 ng of p24 gag antigen) strain of HIV-1 obtained from the National Institutes of Health (NIH) AIDS Research and Reference Reagent Program. After 8 h of infection, cells were washed and fresh media was added. Samples were collected every sixth day and stored at -20°C for p24 gag enzyme-linked immunosorbent assay (ELISA). For HIV-1 p24 ELISA, media from infected cell lines was centrifuged to pellet the cells and supernatants were collected and diluted to 1:100 to 1:1,000 in RPMI 1640 prior to analysis. Supernatants from the infected PBMC were collected and used directly for the p24 antigen assay. The p24 gag antigen level was analyzed using the HIVAG-1 Monoclonal Antibody Kit (Abbott Laboratories, Diagnostics Division).

### siRNA analysis

siRNA sequences were designed using the Oligoengine Workstation  and were purchased from Qiagen-Xeragon. Candidate sequences were chosen based on general siRNA design criteria, including a %GC content between 45–55 % and avoiding more than three consecutive guanosines. Selected target sequences were also BLASTed  with a standard nucleotide-nucleotide BLAST to ensure they were not homologous to other genes. Each candidate siRNA was generated from the 5' end and consisted of 19 nucleotides with a d(TT) overhang.

The following genes were chosen for siRNA analysis with the GenBank accession numbers in brackets: HIV-1 Rev-binding protein 2 [U00943], Pou2AF1 (OBF1) [Z49194], cyclin A1 [U66838], PPGB [NM_000308], cdk2 [AF512553], cdk9 [AF517840], EXT2 [U67368], and HEXA [M16424]. 2 candidate siRNAs were chosen for each of the 8 genes to ensure protein expression silencing. For each duplex siRNA, the first sequence represents the sense sequence ("s"), and the second, the antisense sequence ("as"):

#### HIV-1 Rev-binding protein 2

1. s: GGUCCAAUGGCUGAAACUG, as: CAGUUUCAGCCAUUGGACC

2. s: ACAGUCAUGCUGCCUUCGA, as: UCGAAGGCAGCAUGACUGU

#### Pou2AF1 (OBF-1)

1. s: GAGGAUAGCGACGCCUAUG, as: CAUAGGCGUCGCUAUCCUC

2. s: UGUCACGACAAGAAGCUCC, as: GGAGCUUCUUGUCGUGACA

#### Cyclin A1

1. s: ACUGCAGCUCGUAGGAACA, as: UGUUCCUACGAGCUGCAGU

2. s: GUAGACACCGGCACACUCA, as: UGAGUGUGCCGGUGUCUAC

#### PPGB

1. s: CUAAUGACACUGAGGUCGC, as: GCGACCUCAGUGUCAUUAG

2. s: UGCGUGACCAAUCUUCAGG, as: CCUGAAGAUUGGUCACGCA

#### Cdk2

1. s: AUCCGCCUGGACACUGAGA, as: UCUCAGUGUCCAGGCGGAU

2. s: UCCUCCUGGGCUGCAAAUA, as: UAUUUGCAGCCCAGGAGGA

#### Cdk9

1. s: CCACGACUUCUUCUGGUCC, as: GGACCAGAAGAAGUCGUGG

2. s: CCGCUGCAAGGGUAGUAUA, as: UAUACUACCCUUGCAGCGG

#### EXT2

1. s: GCACCUCGAGCUAUGCAAC, as: GUUGCAUAGCUCGAGGUGC

2. s: CUCCGUCUUUGGCCUGACA, as: UGUCAGGCCAAAGACGGAG

#### HEXA

1. s: CCUGGUCACAAAAGAGCCU, as: AGGCUCUUUUGUGACCAGG

2. s: GUGUGAAUGGCGUUAGGGU, as: ACCCUAACGCCAUUCACAC

HIV-1 latently infected OM-10.1 T lymphocytes were treated with 10 μg of the various siRNAs listed above for 48 hrs prior to TNF-α treatment. siRNAs were electroporated into OM-10.1 cells at 5 × 10^6 ^(mid log phase of growth) cells/ml. 48 hrs later cells were treated with TNF-α (5 μg/ml for 2 hrs) to induce viral transcription and progeny formation, washed, and complete media was added to cells. Samples were collected at 72 hrs post-TNF-α treatment for presence of HIV-1 p24 Gag by ELISA. Presence of p24 Gag in the supernatant is indicative of mature infectious virion particles released from HIV-1 infected cells.

### Expression profiling

Six μg of cytoplasmic RNA from each sample were converted to double-stranded cDNA using the Superscript Choice System kit and T7-(dT)24 primer (100 pmol/μL) (Invitrogen). The cDNA was cleaned and purified using phenol/chloroform extraction and ethanol precipitation. The cDNA was then used to perform *in vitro *transcription using the BioArray HighYield RNA Transcript Labeling Kit (T7) (Enzo, Farmingdale, NY). The biotin-labeled cRNA was cleaned using the RNeasy Mini Kit (Qiagen) and was quantified by spectrophotometric analysis and analyzed on a 1% agarose TAE gel. The biotin-labeled cRNA was then randomly fragmented to ~35–200 base pairs by metal-induced hydrolysis using a fragmentation buffer according to the Affymetrix Eukaryotic Target Hybridization protocol. The Human U95Av2 microarrays (Affymetrix) were washed, primed, and stained on the Affymetrix Fluidics Station 400 following the Affymetrix protocol. cRNA was first detected through a primary scan with phycoerythrin-streptavidin staining and then amplified with a second stain using biotin-labeled anti-streptavidin antibody and a subsequent phycoerythrin-streptavidin stain. The emitted fluorescence was scanned using the Hewlett-Packard G2500A Gene Array Scanner, and the intensities were extracted from the chips using Microarray Suite 4.0 (MAS4.0) software. All raw chip data was scaled in MAS4.0 to 800 to normalize signal intensities for inter-array comparisons. A statistical algorithm in MAS4.0 assigns present, marginal, and absent calls based on probe pair intensities where one probe is a perfect match of a reference sequence and the other is a mismatch probe that has a single base change at the 13th position within the 25-base oligonucleotide reference sequence.

### Quality Control

Report files generated by MAS4.0 were reviewed to ensure all quality control standards were met – these include percentage of present calls, presence of spike controls, signal scaling factors per chip, and the GAPDH 3'/5' ratios. All raw data files containing the signal and detection values for each probe set and supplemental data files are posted on a Translational Genomics (TGen) data site, , as well as on the Gene Expression Omnibus (GEO) online repository  as identified by GEO accession number [see Additional File 1].

### Data analysis

Comparative analyses were performed in MAS4.0 between replicate samples to determine gene expression behavior changes between every sample set; calls assigned by MAS4.0 can be either increase, marginally increase, decrease, marginally decrease, or no change.

Comprehensive microarray data analysis was performed using GeneSpring software (v4.2; Silicon Genetics, Redwood City, CA). Using the synchronized cell cycle data, a gene list was generated by filtering for genes that had (1) a minimum of 2 present calls (detection as determined by MAS4.0) out of a total of 32 calls (1 call per chip), (2) a maximum p-value of 0.05 where, in this case, the p-value represents the probability that the signal intensity for a gene is due to chance alone, and (3) a greater than 2-fold expression change between control pCep4 samples and respective eTat samples. To divide the genes in this list into groups based on similar expression patterns through the cell cycle, k-means clustering (of 15 clusters as selected based on Genespring's expressed validity value) was applied and gene lists for each cluster were consolidated [see Additional Files 3 and 7].

A complementary analysis was also performed using SOMs [54]. The input gene list for this analysis was generated using several filters against the entire list of probe sets, which represent the gene transcripts on the U95Av2 array: (1) filter for at least 2 present calls, (2) any probe sets that generated an absent call across all cell cycle time points were eliminated, (3) any probe sets that did not have three out of four marginal increase or increase calls, or marginal decrease or decrease calls in at least one of the eight cell cycle time points, were removed (based on comparative analyses generated by MAS4.0) to control for replicate consistency. The signal log ratio of each gene in the resulting list was calculated (using the two replicate eTat samples and 2 replicate pCep4 samples per time point for each gene):



Three sets of gene lists were created based on 3 separate filtering rules:

(1) 0.666 < ratio < 1.500

(2) 0.500 < ratio < 2.000

(3) 0.333 < ratio < 3.000

For a single rule, if a gene had average signal ratios at every time point that fell within the specified boundary, the gene was removed from the list. Separate gene lists were generated for each rule. For the first rule, 464 genes were removed and 2330 genes were used for clustering; the second rule, 1644 genes were removed and 1150 were used for analysis; and for the third rule, 2415 genes were eliminated and 379 were used for clustering. The gene ratios in each of the three lists were log transformed (natural base), median centered, applied to separate SOMs, and visualized using the U-matrix and component planes representation [for each SOM see Additional Files 5 and 6, and Figure 4, respectively] [54,55]. The algorithm incorporates a batch learning algorithm with Euclidean distance, and all computations were performed using MATLAB (The MathWorks) with the SOM-toolbox with parameters set to defaults as described [56]. Defined groups of neurons that displayed expression differences from one time point to the next in the component planes representation, as well as clusters appearing in the U-matrix were noted. Neurons in the same position across the component planes contain the same genes; thus, coloring of the neurons allows for direct interpretation of the differences in expression levels between time points. Gene lists corresponding to the first and third filters were consolidated [see Additional File 2].

The original gene list of synchronized sample data was also filtered for those genes that had all absent calls in the control cells and at least 2 present calls in the experimental cells. The resulting gene list was surveyed against 540 Affymetrix Hu95 chips whose data is hosted at the Children's National Medical Center (CNMC) in Washington, D.C. . These human data include all control and experimental data produced from the study of different genetic diseases in a variety of human tissues and cultured cells. Those genes from our gene lists that were 100% absent or 50.1% to 99.9% absent across all Hu95 data in the database were compiled and noted to provide an estimate of the drug target specificity.

### Gene classification/ontologies

Genes were classified as functionally relevant to HIV-1 after exhaustive literature review of publications indexed on the Entrez PubMed website. Affymetrix probe set identifiers from the increasing and decreasing expression lists were queried on the Affymetrix website  using the NetAffx analysis tool to determine gene names and functions. The genes in the resulting lists were classified into ontologies to show the genes having increased or decreased expression (organized based on their respective functions). For the gene ontology for the entire human U95Av2 genechip, ontology lists specific to the classifications available on Genespring v5.0.3 were first obtained. The remaining classifications were queried on the Affymetrix website with the NetAffx tool .

## Abbreviations

HIV-human immunodeficiency virus

PBMC-peripheral blood mononuclear cells

HAART-highly active retroviral therapy

NNRTI-non-nucleoside reverse transcriptase inhibitor

NRTI-nucleoside reverse transcriptase inhibitor

TAR-transactivation response

Hu-hydroxyurea

Noco-nocodazole

CNMC-Children's National Medical Center

NA-neuraminidase

FGFR-fibroblast growth factor receptor

EGFR-epidermal growth factor receptor

ELISA-enzyme-linked immunosorbent assay

## Competing Interests

The author(s) declare that they have no competing interests.

## Authors' Contributions

WSL performed the data analyses and helped to draft the manuscript. AM, and EA performed the siRNA experiments, coordinated data analysis, and helped to draft the manuscript. TT performed the expression profiling protocol on all samples. CdlF isolated RNA and contributed to the expression profiling experiment. KK, CdlF, and SD helped with the gene expression profiling, westerns, and FACS. SH ran the self-organizing map analyses. AP provided some of the supervision for the manuscript and support for the Kashanchi lab members. DAS coordinated the expression profiling and analytical methodology. FK participated in the design, coordination, and validation of the study. DAS and FK funded the studies. All authors have read and approved the manuscript.

## Supplementary Material

Additional File 5 Self-organizing map (SOM) for filter 1 (refer to Methods) Click here for file

Additional File 6 Self-organizing map (SOM) for filter 2 (refer to Methods) Click here for file

Additional File 7 K-means clustering (15 graphs and corresponding close-ups shown) Click here for file

Additional File 4 Gene lists filtered for all absent in pCep4 samples and at least 2 present calls in eTat samples (Excel worksheet, "2P"), 4 present calls in eTat samples (Excel worksheet, "4P"), and 8 present calls in eTat samples (Excel spreadsheet, "8P") Click here for file

Additional File 1 GEO accession numbers for each sample Click here for file

Additional File 3 K-means clustering gene lists (three Excel worksheets, "set1–5," "set6–10," "set11–15") Click here for file

Additional File 2 Self-organizing map (SOM) gene lists for the first and third filters (two Excel worksheets, "HIV_SOM_Filt_1a" & "HIV_SOM_Filt_3a") Click here for file
