# Supplementary figures and images for: Therapeutic targets for HIV-1 infection in the host proteome
Source: Retrovirology. 2005 Mar 21;2:20. doi: 10.1186/1742-4690-2-20 (PMC1087880; doi:10.1186/1742-4690-2-20)

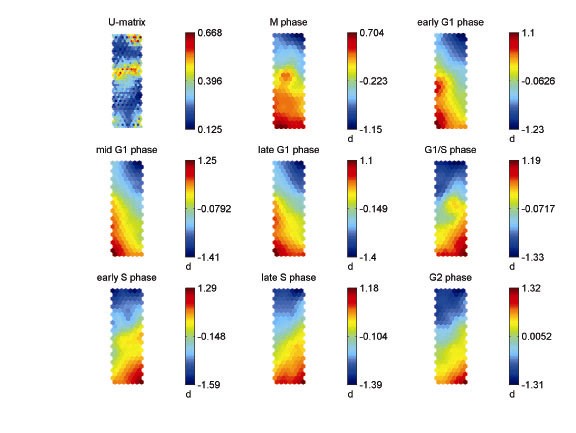

Supplement: Additional File 5 — Self-organizing map (SOM) for filter 1 (refer to Methods) [file 1742-4690-2-20-S5.jpeg]
