# Supplementary material for: Therapeutic targets for HIV-1 infection in the host proteome
Source: Retrovirology. 2005 Mar 21;2:20. doi: 10.1186/1742-4690-2-20 (PMC1087880; doi:10.1186/1742-4690-2-20)
Supplement: Additional File 7 — K-means clustering (15 graphs and corresponding close-ups shown) [file 1742-4690-2-20-S7.ppt]

## Slide 1
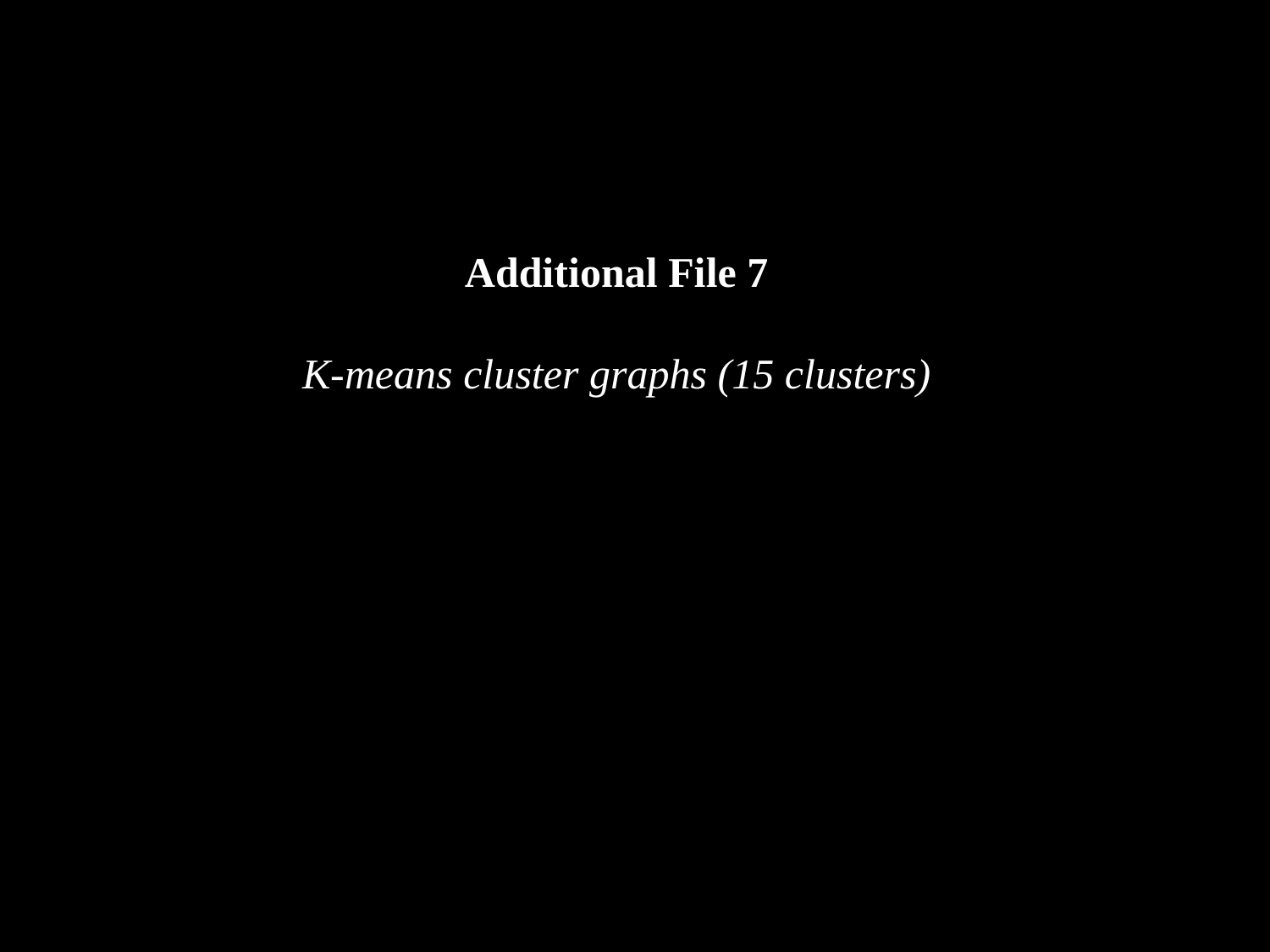

Additional File 7
K-means cluster graphs (15 clusters)

## Slide 2
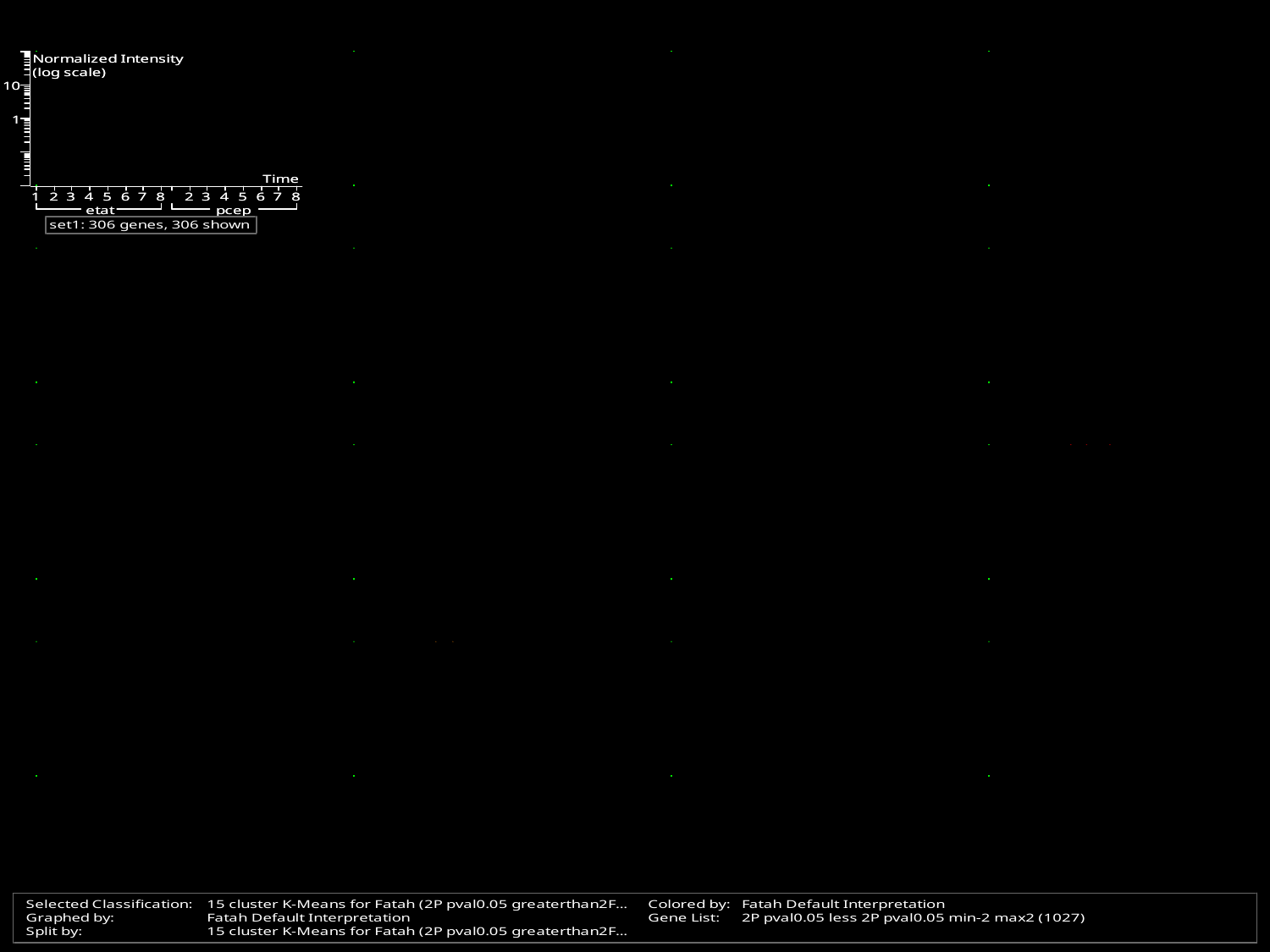

## Slide 3
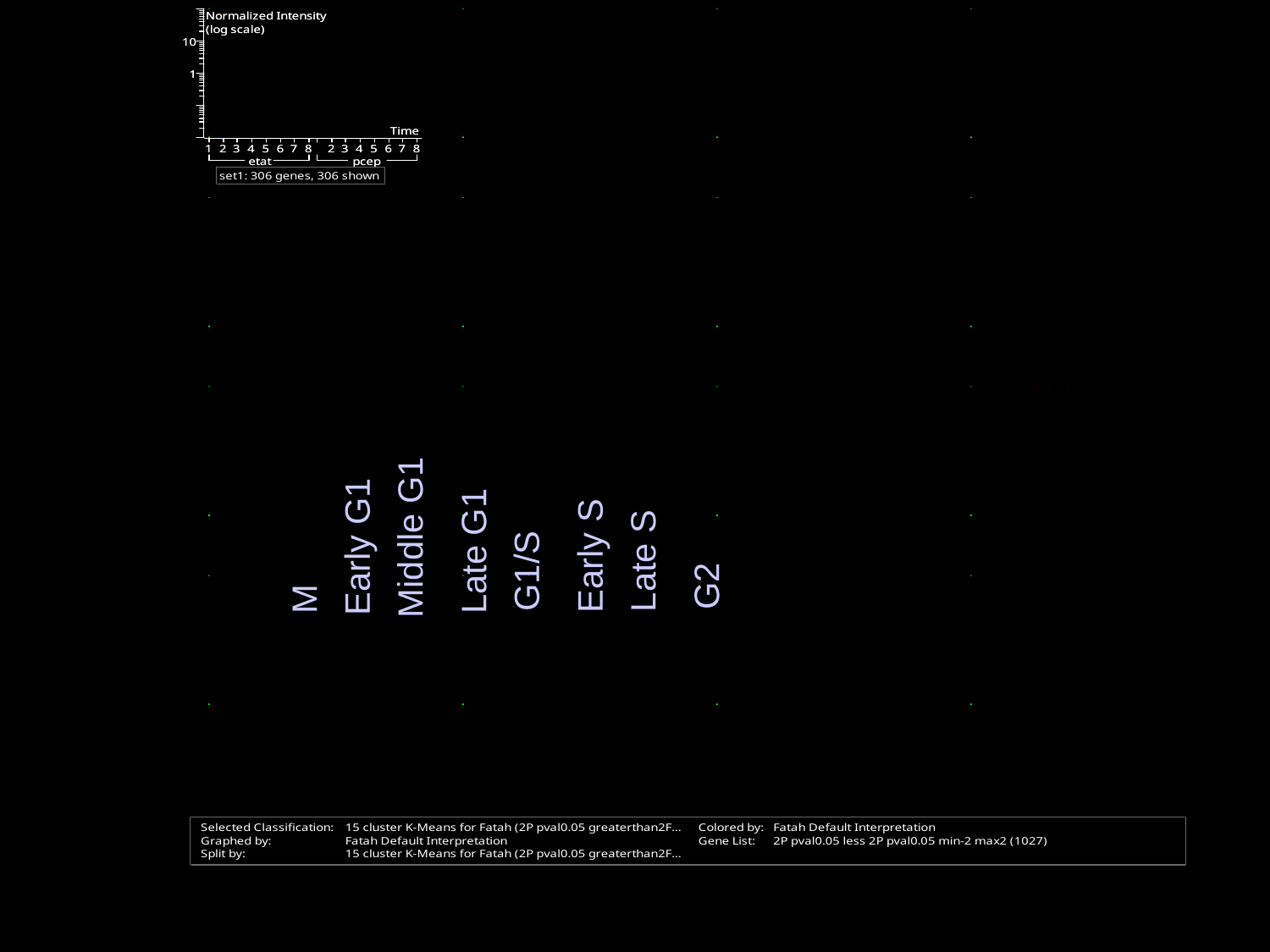

Middle G1
Early G1
Late G1
Early S
Late S
G1/S
G2
M

## Slide 4
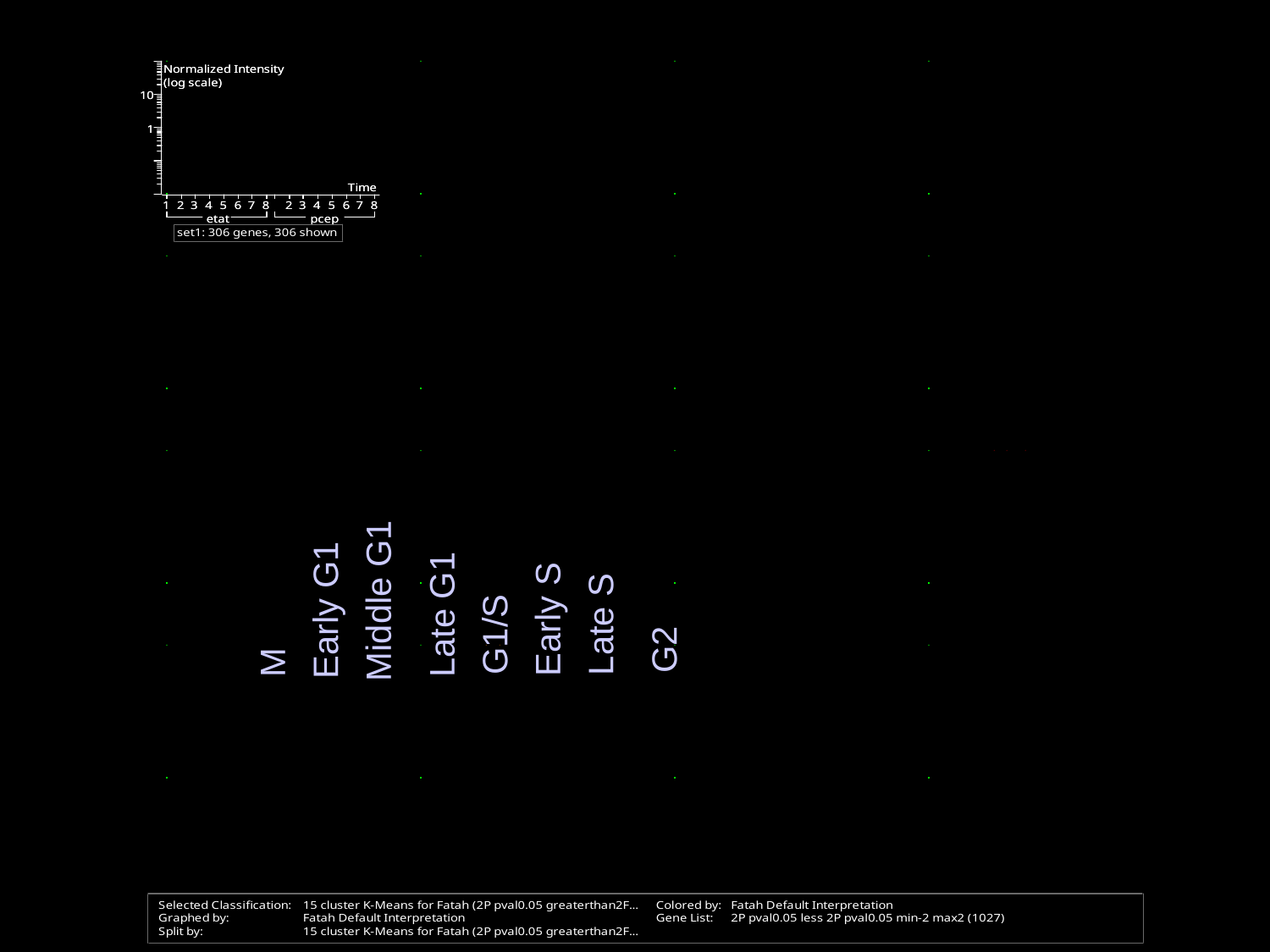

Middle G1
Early G1
Late G1
Early S
Late S
G1/S
G2
M

## Slide 5
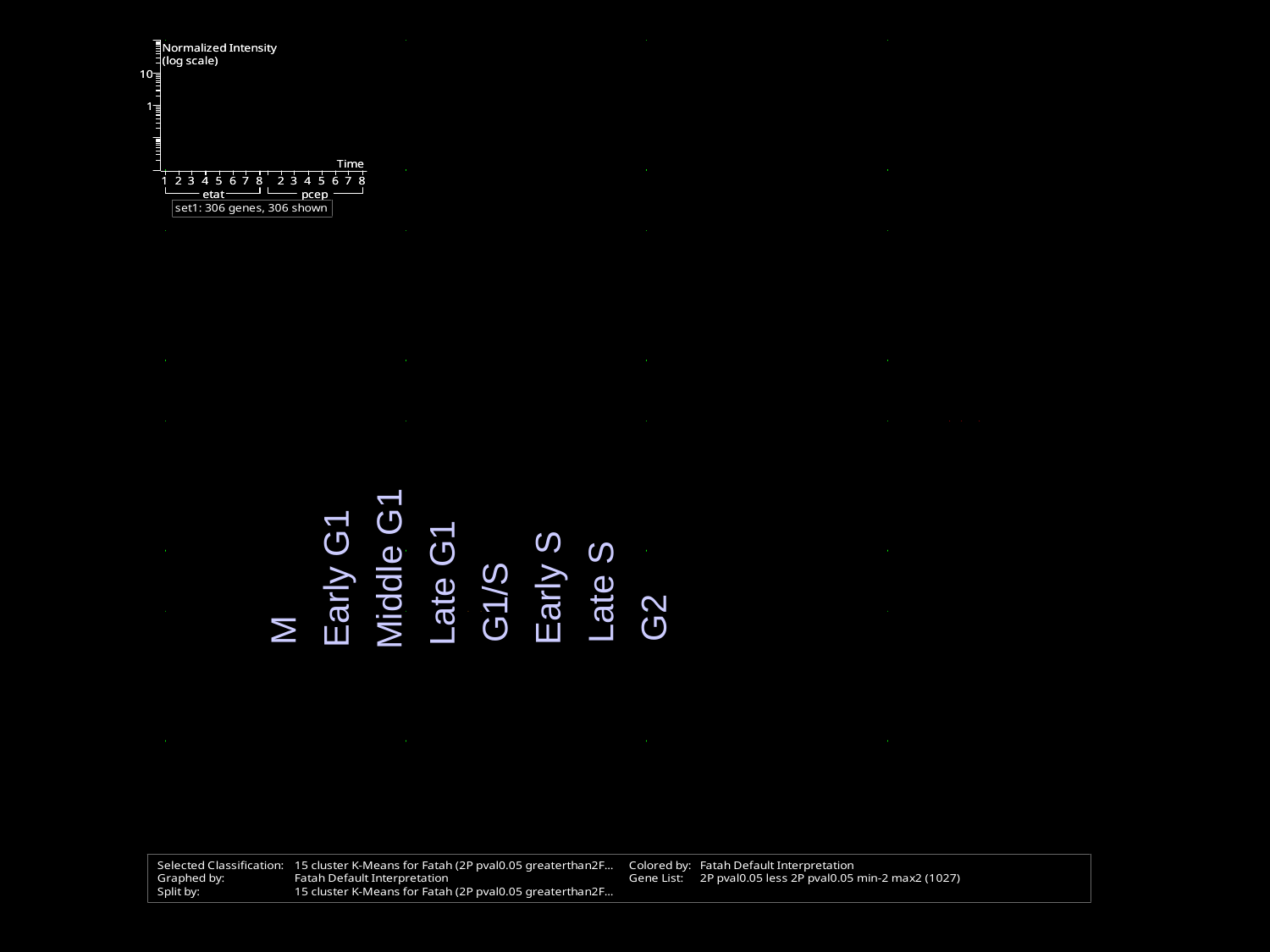

Middle G1
Early G1
Late G1
Early S
Late S
G1/S
G2
M

## Slide 6
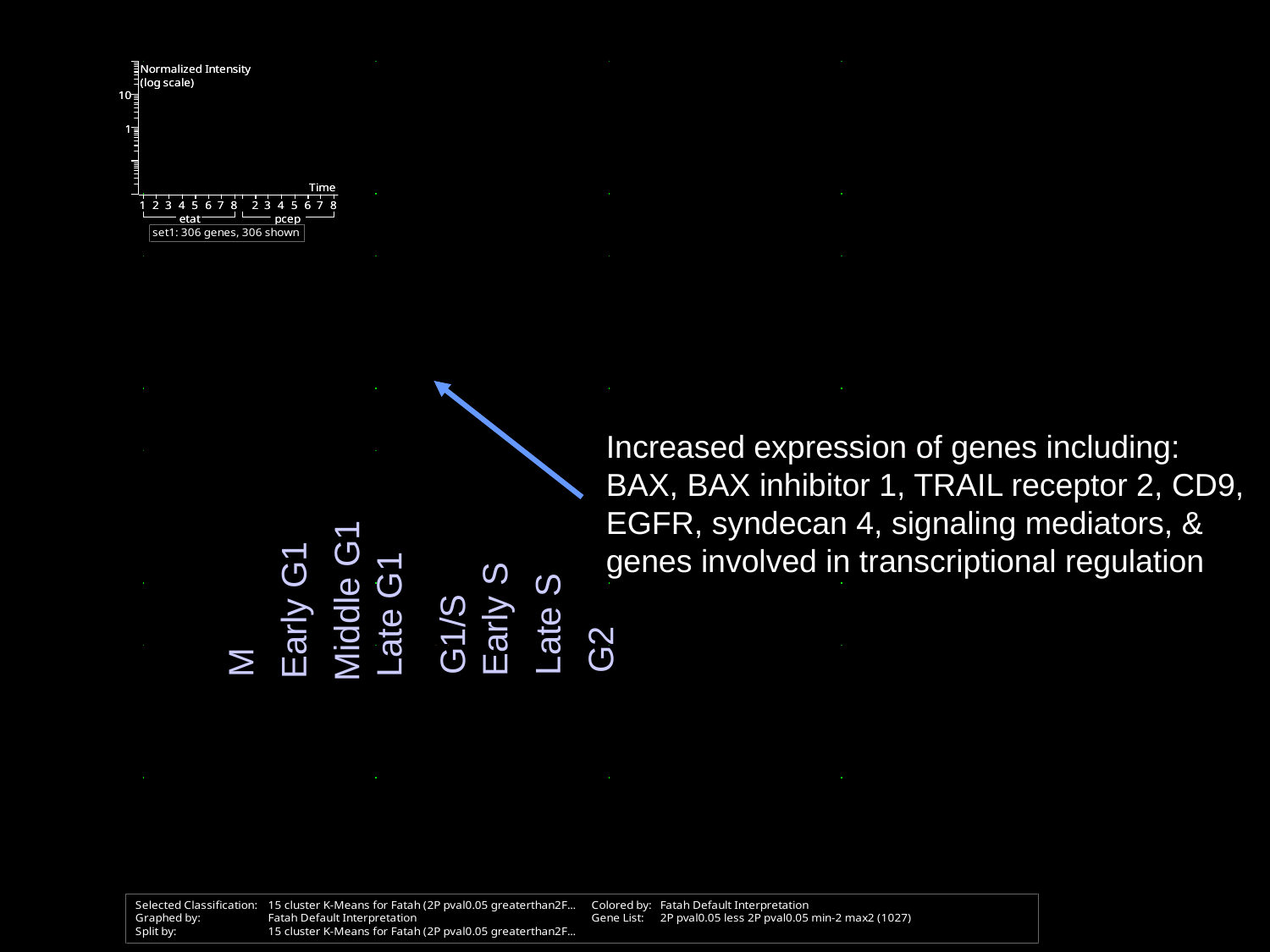

Increased expression of genes including:
BAX, BAX inhibitor 1, TRAIL receptor 2, CD9,
EGFR, syndecan 4, signaling mediators, &
genes involved in transcriptional regulation
Middle G1
Early G1
Late G1
Early S
Late S
G1/S
G2
M

## Slide 7
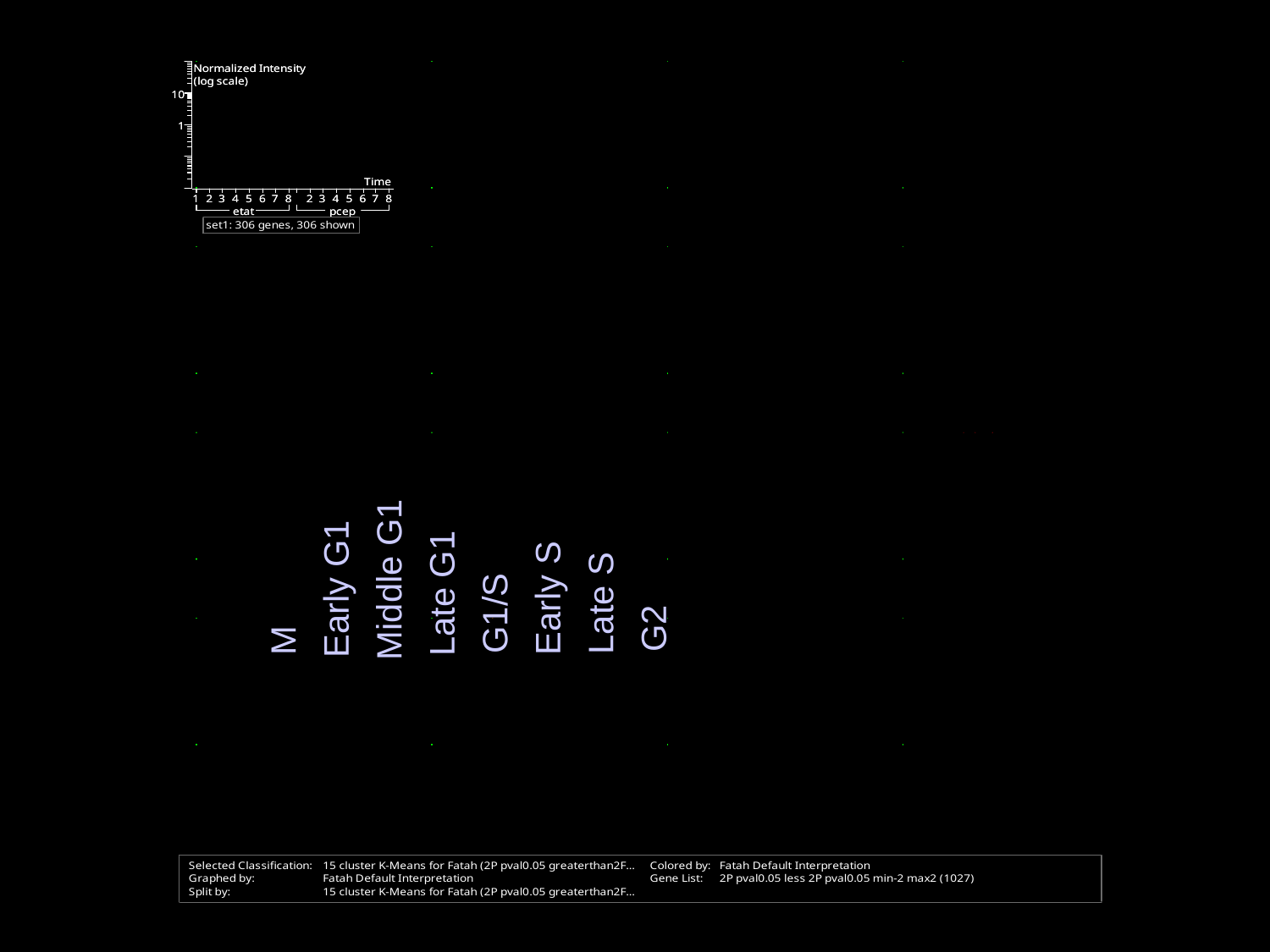

Middle G1
Early G1
Late G1
Early S
Late S
G1/S
G2
M

## Slide 8
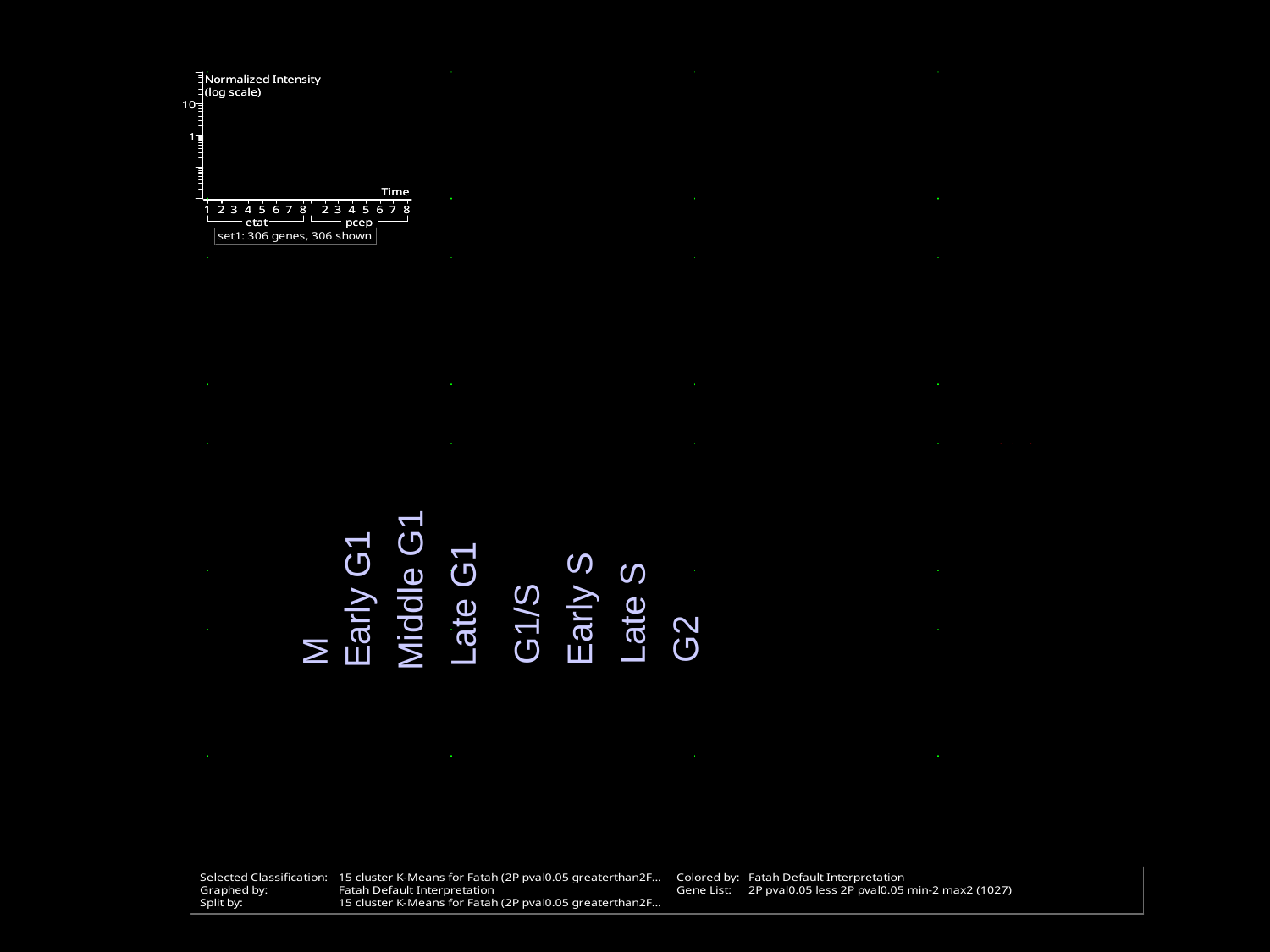

Middle G1
Early G1
Late G1
Early S
Late S
G1/S
G2
M

## Slide 9
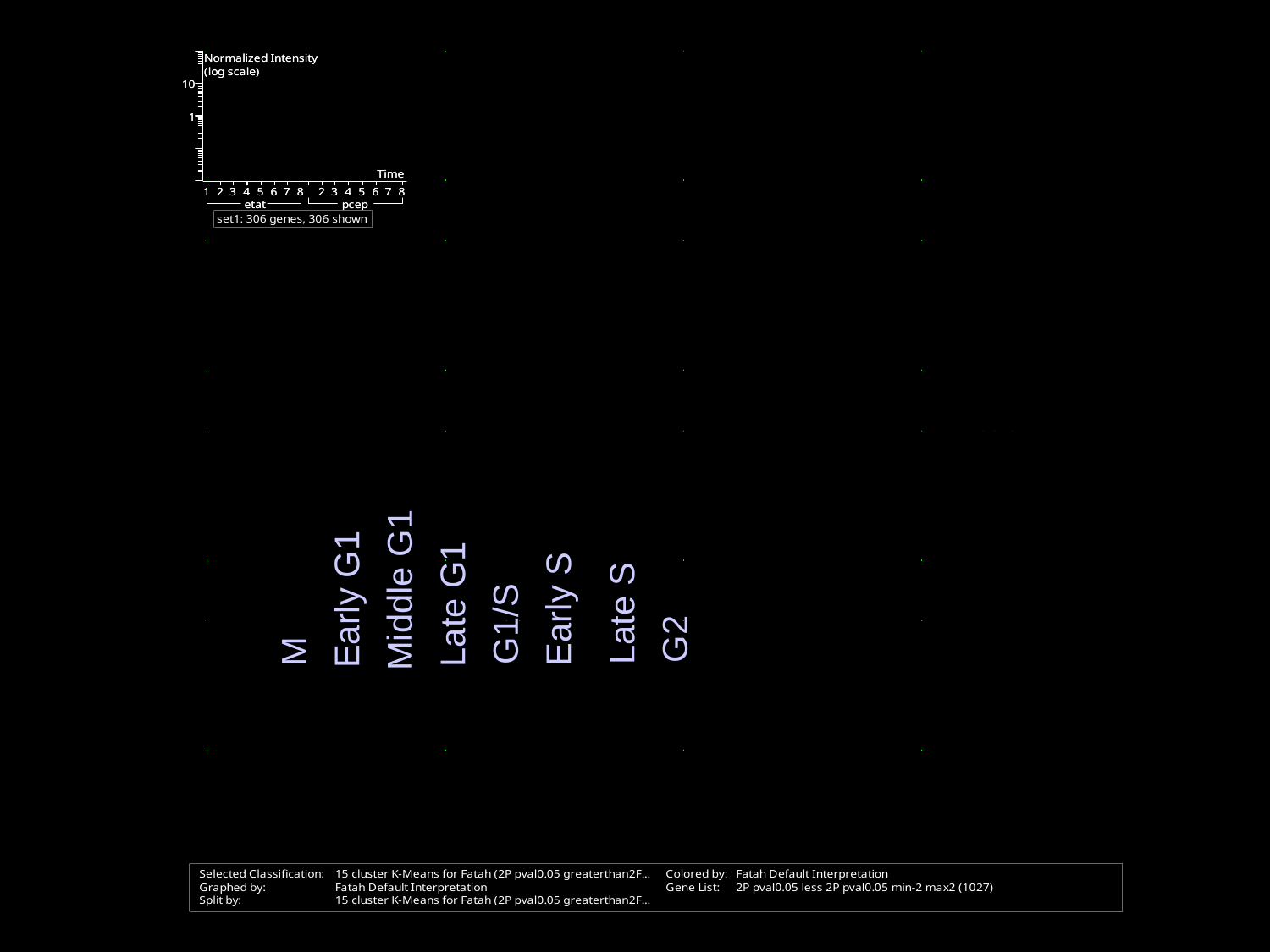

Middle G1
Early G1
Late G1
Early S
Late S
G1/S
G2
M

## Slide 10
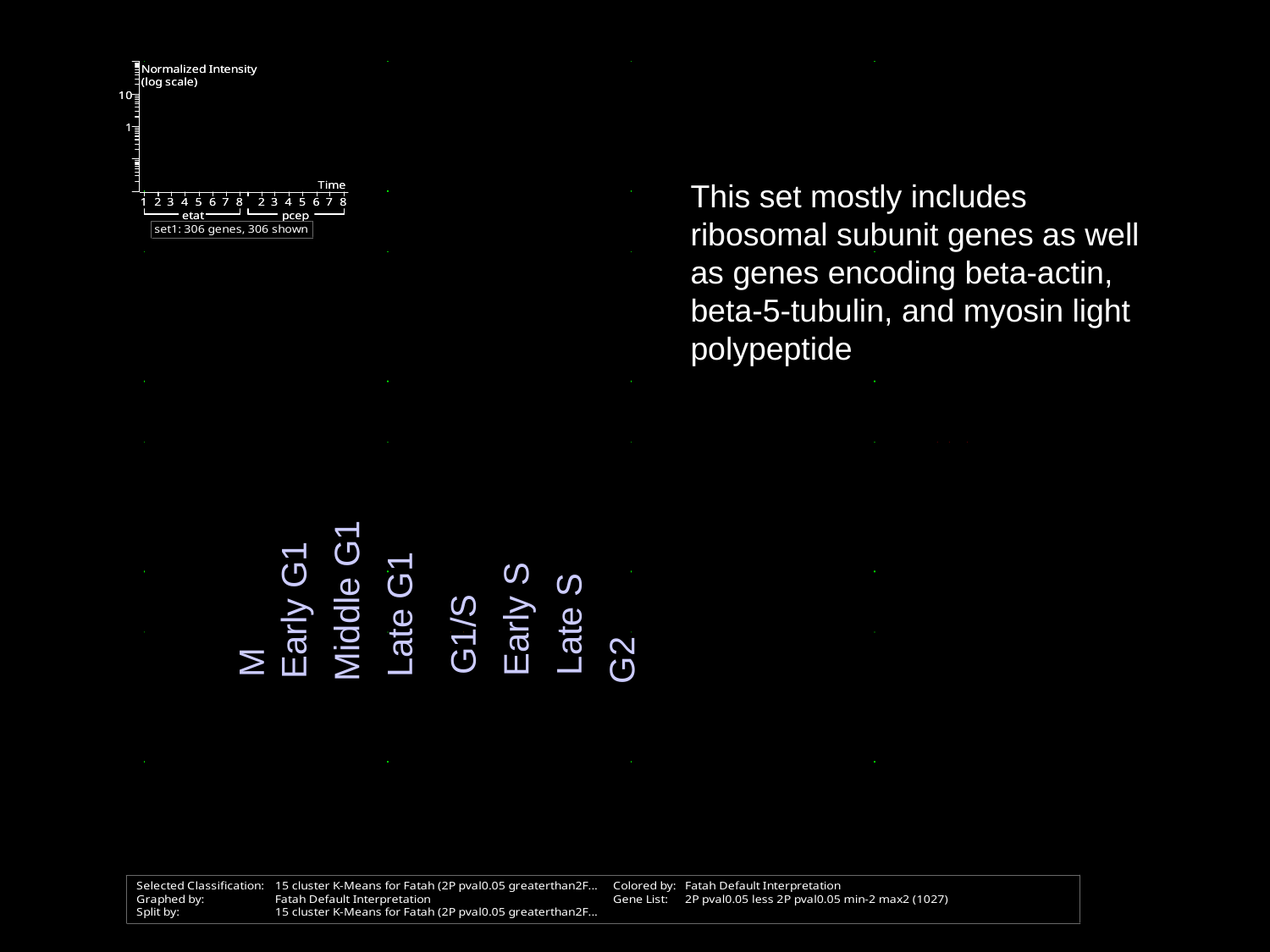

This set mostly includes
ribosomal subunit genes as well
as genes encoding beta-actin,
beta-5-tubulin, and myosin light
polypeptide
Middle G1
Early G1
Late G1
Early S
Late S
G1/S
G2
M

## Slide 11
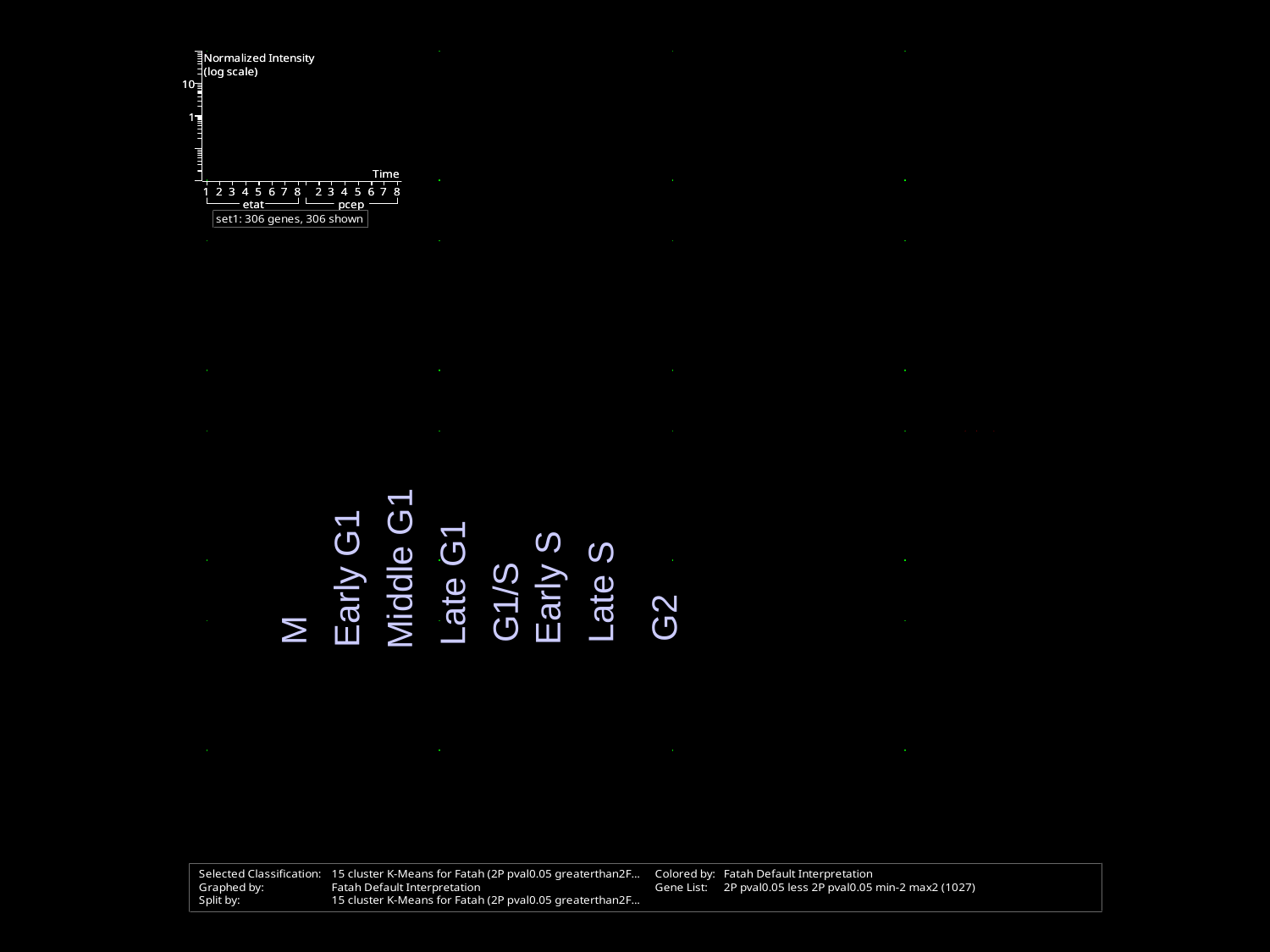

Middle G1
Early G1
Late G1
Early S
Late S
G1/S
G2
M

## Slide 12
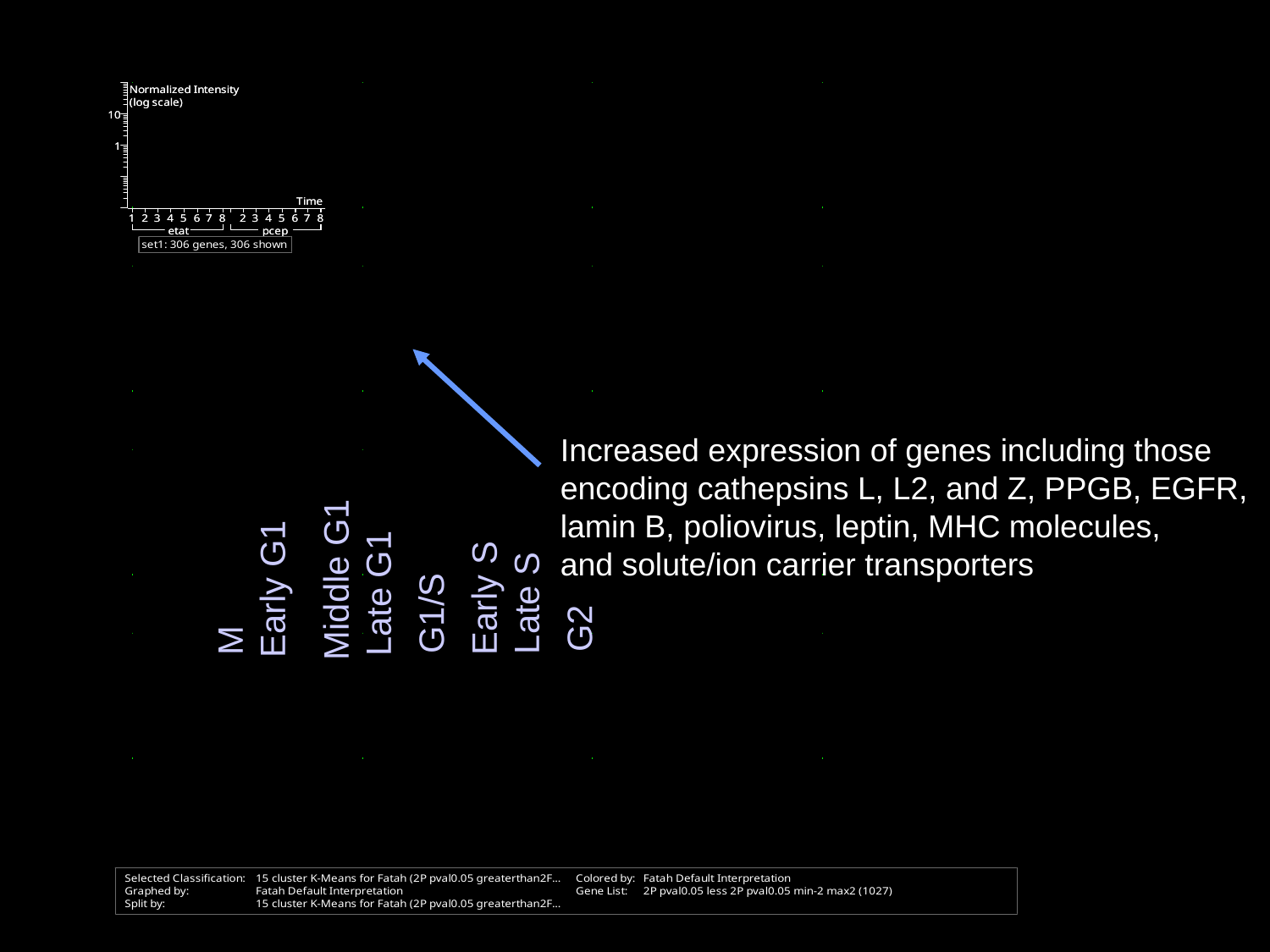

Increased expression of genes including those
encoding cathepsins L, L2, and Z, PPGB, EGFR,
lamin B, poliovirus, leptin, MHC molecules,
and solute/ion carrier transporters
Middle G1
Early G1
Late G1
Early S
Late S
G1/S
G2
M

## Slide 13
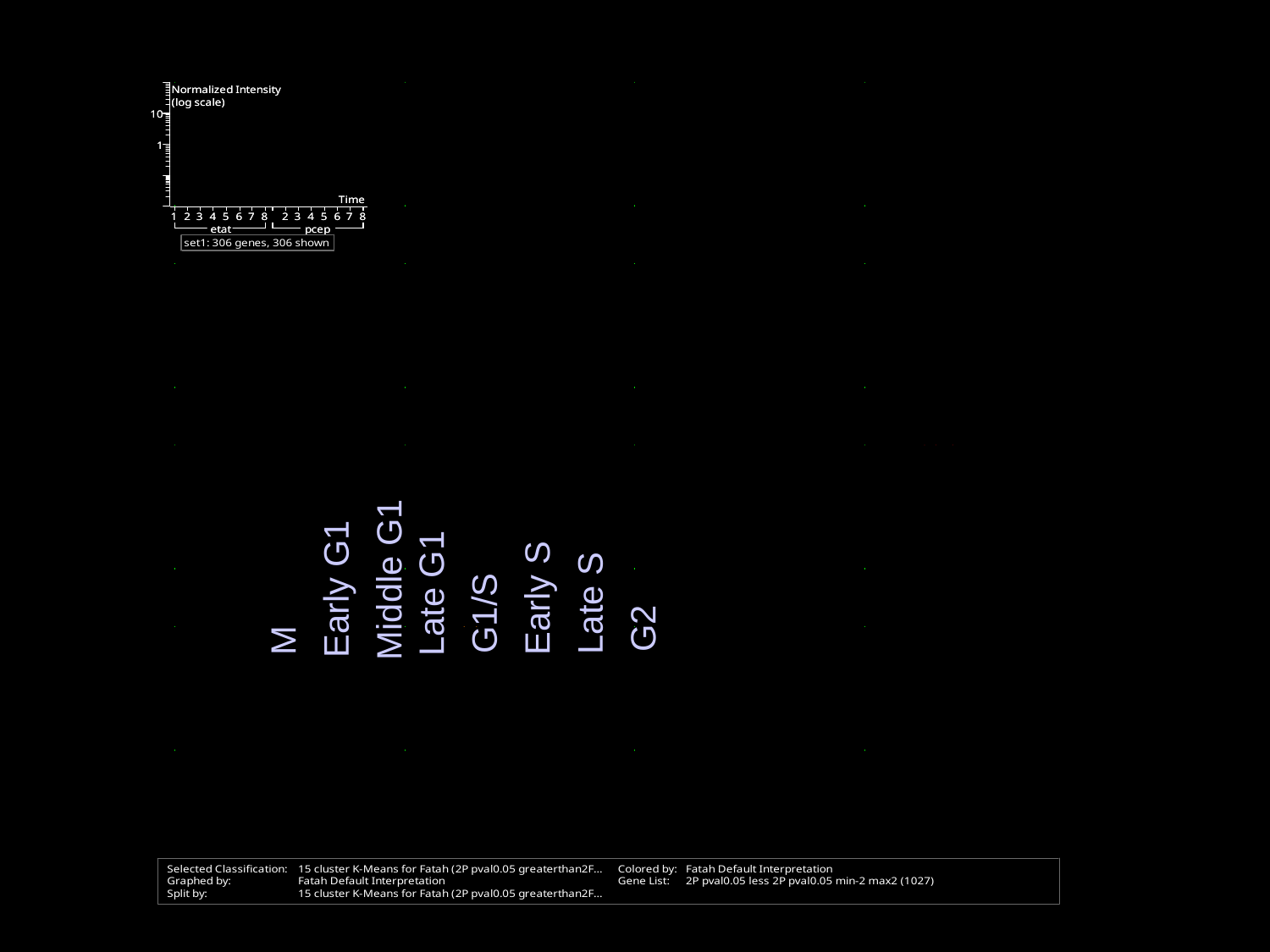

Middle G1
Early G1
Late G1
Early S
Late S
G1/S
G2
M

## Slide 14
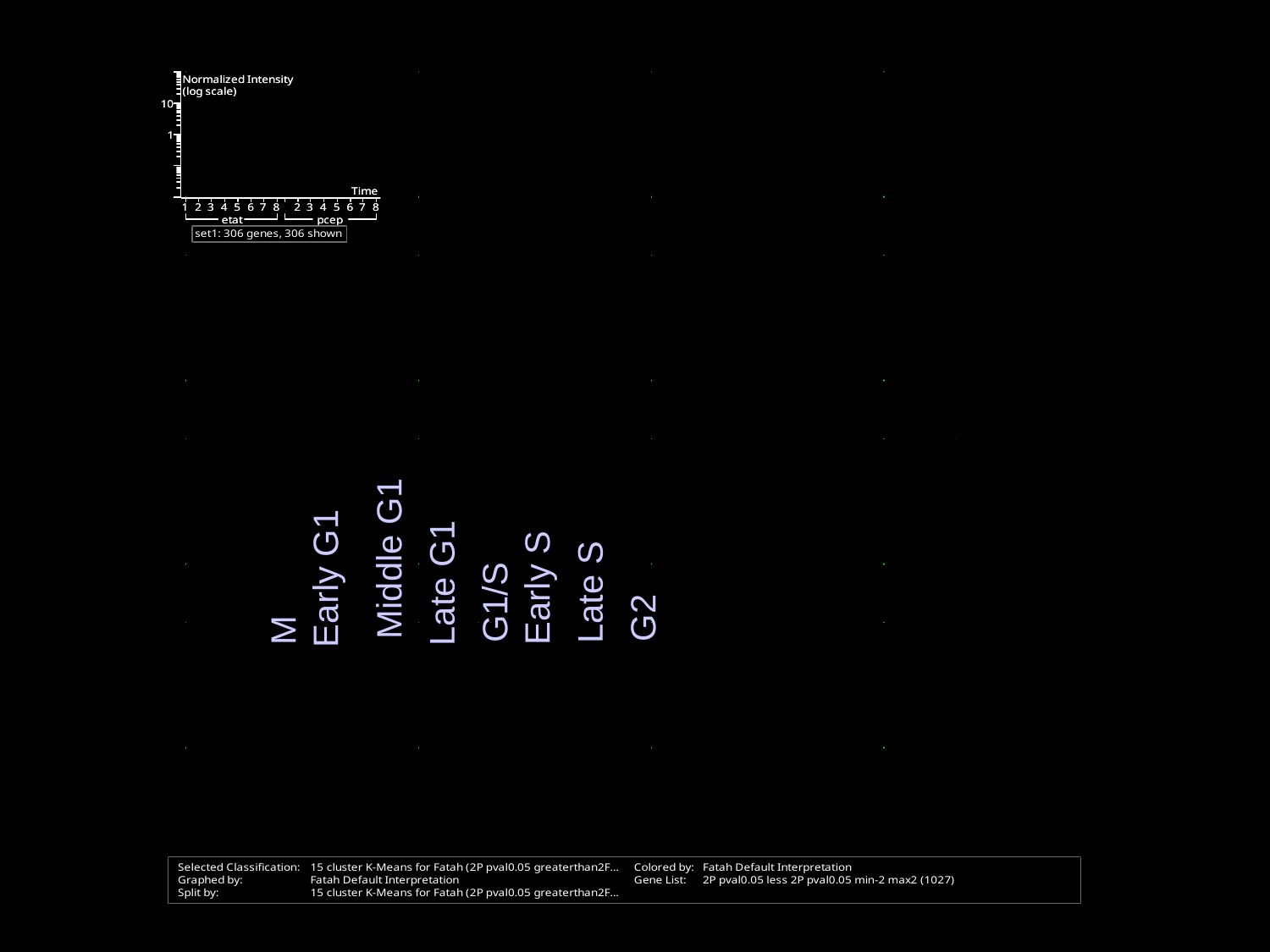

Middle G1
Early G1
Late G1
Early S
Late S
G1/S
G2
M

## Slide 15
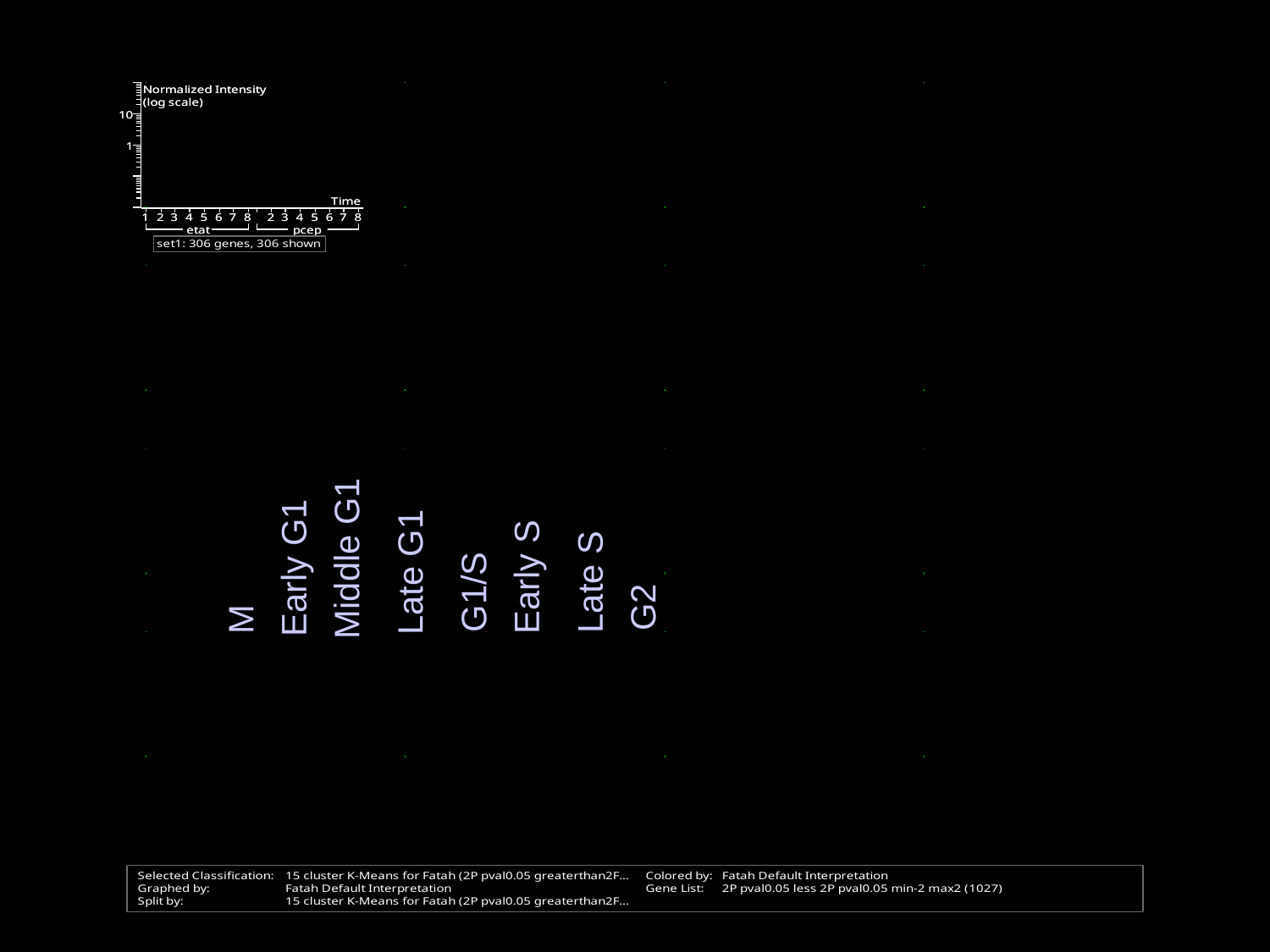

Middle G1
Early G1
Late G1
Early S
Late S
G1/S
G2
M

## Slide 16
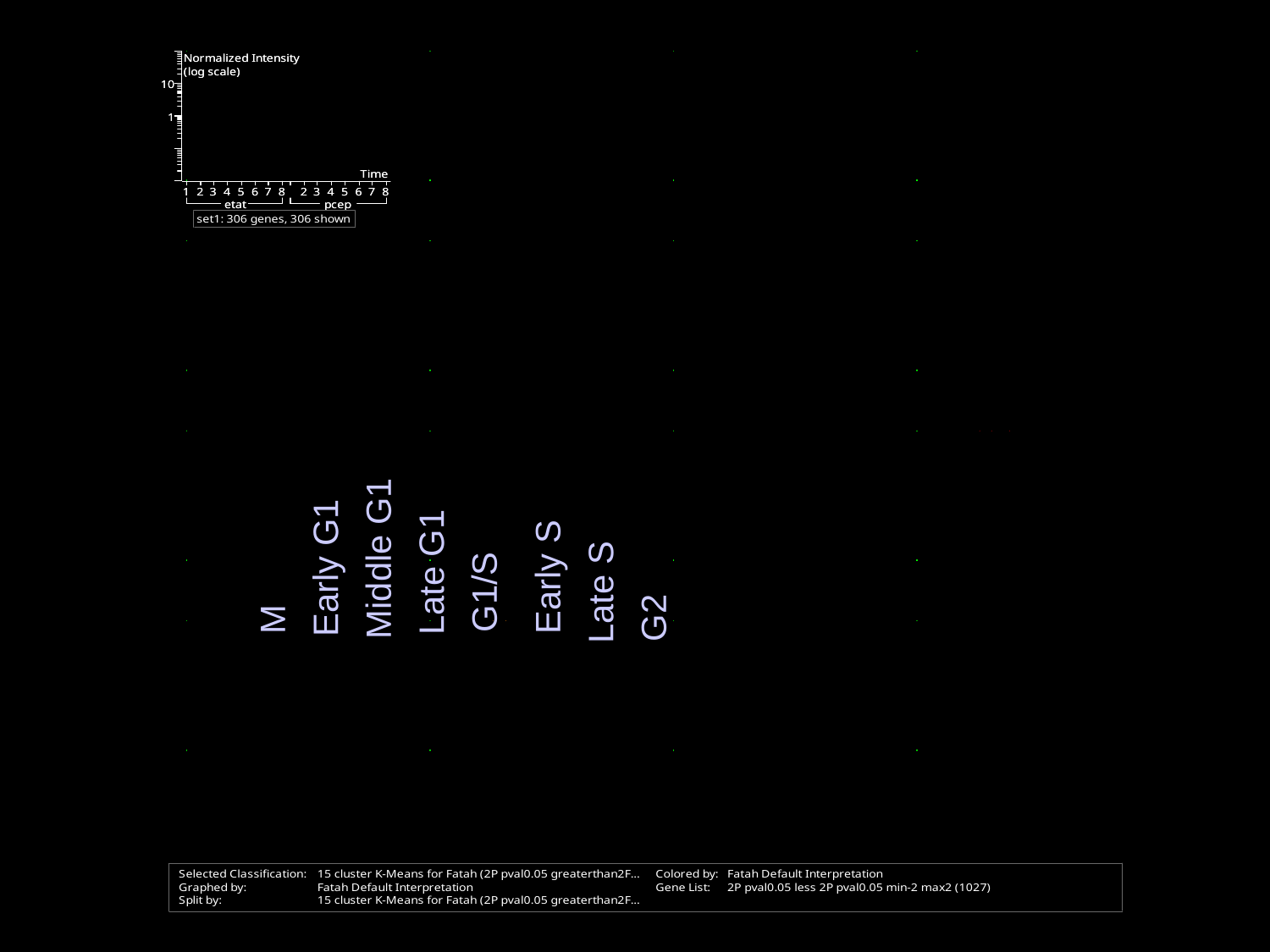

Middle G1
Early G1
Late G1
Early S
G1/S
Late S
G2
M

## Slide 17
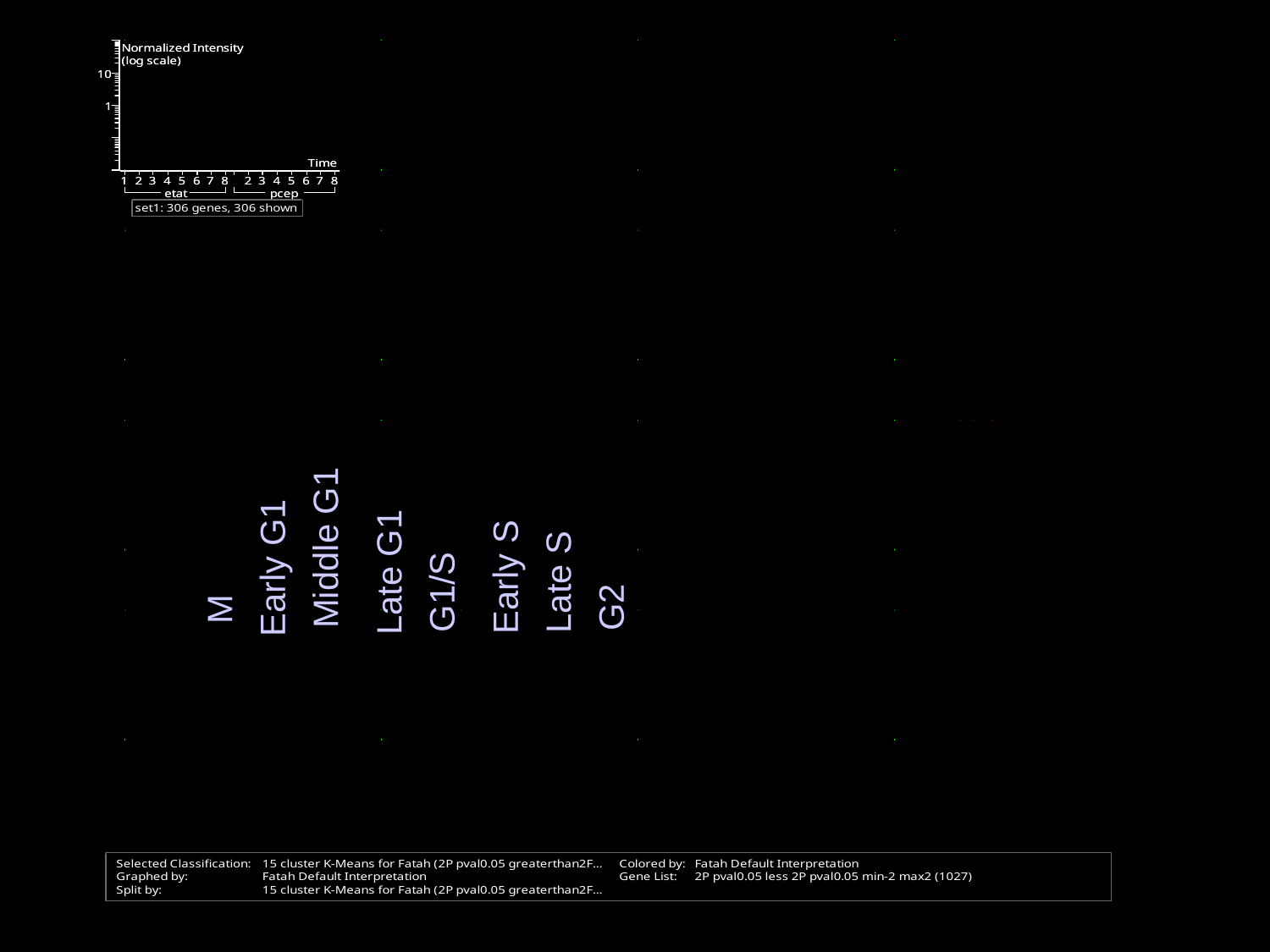

Middle G1
Early G1
Late G1
Early S
Late S
G1/S
G2
M
